# Low-Dose Recombinant Adeno-Associated Virus-Mediated Inhibition of Vascular Endothelial Growth Factor Can Treat Neovascular Pathologies Without Inducing Retinal Vasculitis

**DOI:** 10.1089/hum.2021.132

**Published:** 2021-07-19

**Authors:** Shun-Yun Cheng, Yongwen Luo, Anneliese Malachi, Jihye Ko, Qin Su, Jun Xie, Bo Tian, Haijiang Lin, Xiao Ke, Qiang Zheng, Phillip W.L. Tai, Guangping Gao, Claudio Punzo

**Affiliations:** ^1^ Department of Ophthalmology and Visual Sciences, University of Massachusetts Medical School, Worcester, Massachusetts, USA; ^2^ Horae Gene Therapy Center, University of Massachusetts Medical School, Worcester, Massachusetts, USA; ^3^ College of Veterinary Medicine, South China Agricultural University, Guangzhou, China; ^4^ Viral Vector Core, University of Massachusetts Medical School, Worcester, Massachusetts, USA; ^5^ Department of Microbiology and Physiological Systems, University of Massachusetts Medical School, Worcester, Massachusetts, USA; ^6^ Chengdu Kanghong Pharmaceutical Group Co. Ltd, Chengdu, Sichuan, China; ^7^ Li Weibo Institute for Rare Diseases Research, University of Massachusetts Medical School, Worcester, Massachusetts, USA.

**Keywords:** CNV, AMD, DR, wet AMD, VEGF, anti-VEGF, conbercept, rAAV, vasculitis

## Abstract

The wet form of age-related macular degeneration is characterized by neovascular pathologies that, if untreated, can result in edemas followed by rapid vision loss. Inhibition of vascular endothelial growth factor (VEGF) has been used to successfully treat neovascular pathologies of the eye. Nonetheless, some patients require frequent intravitreal injections of anti-VEGF drugs, increasing the burden and risk of complications from the procedure to affected individuals. Recombinant adeno-associated virus (rAAV)-mediated expression of anti-VEGF proteins is an attractive alternative to reduce risk and burden to patients. However, controversy remains as to the safety of prolonged VEGF inhibition in the eye. Here, we show that two out of four rAAV serotypes tested by intravitreal delivery to express the anti-VEGF drug conbercept lead to a dose-dependent vascular sheathing pathology that is characterized by immune cell infiltrates, reminiscent of vasculitis in humans. We show that this pathology is accompanied by increased expression in vascular cell adhesion molecule 1 (VCAM1) and intercellular adhesion molecule 1 (ICAM1), both of which promote extravasation of immune cells from the vasculature. While formation of the vascular sheathing pathology is prevented in immunodeficient Rag-1 mice that lack B and T cells, increased expression of VACM1 and ICAM1 still occurs, indicating that inhibition of VEGF function leads to expression changes in cell adhesion molecules that promote extravasation of immune cells. Importantly, a 10-fold lower dose of one of the vectors that cause a vascular sheathing pathology is still able to reduce edemas resulting from choroidal neovascularization without causing any vascular sheathing pathology and only a minimal increase in VCAM1 expression. The data suggest that treatments of neovascular eye pathologies with rAAV-mediated expression of anti VEGF drugs can be developed safely. However, viral load needs to be adjusted to the tropisms of the serotype and the expression pattern of the promoter.

## Introduction

Age-related macular degeneration (AMD) is the leading cause of blindness in the elderly. It currently affects 196 million people worldwide and is expected to affect 288 million by 2040.^1,2^ The wet form of AMD is a severe advanced form of the disease, characterized by retinal and/or choroidal neovascularization (CNV) that result in retinal edemas, causing rapid vision loss if untreated. Vascular endothelial growth factor (VEGF) is a growth factor that promotes neovascularization and abnormal angiogenesis in multiple vascular-related diseases. Inhibition of VEGF function has been shown to reduce neovascular pathologies and edemas in the eye. Consequently, different anti-VEGF therapies have been developed to treat neovascularization, in particular in AMD and diabetic retinopathy.^3^ However, anti-VEGF drugs require repeat injections to maintain stable and long-term treatment efficacy. Some patients require quite frequent injections, which increases the risk of side effects, such as intraocular hemorrhage and ocular hypertensions.^3,4^ In addition, the burden to patients who require such intense injection regimens is high.

Recombinant adeno-associated virus (rAAV)-directed gene therapy is a propitious treatment for tackling mutation-related problems in inherited diseases.^5^ Despite its wide use for such diseases, rAAV vector-based gene delivery is also ideal for treating nongenetic disorders. Using rAAV vectors to deliver a transgene that can inhibit VEGF function can reduce the burden to patients and the risk of side effects related with other treatment approaches. Consequently, several adenoviral-^6–8^ as well as rAAV-^9–13^ based anti-VEGF therapies have already been developed and tested in various animal models,^14^ some of which have already been evaluated in clinical trials^15–18^ or are currently ongoing^18^ (NCT04514653, NCT04704921, NCT04567550, NTC04418427). All these trials demonstrated efficacious viral delivery of an anti-VEGF protein to neutralize native VEGF and reduce retinal edemas.^18^ However, in a small portion of patients, intraocular inflammation was seen after intravitreal delivery of the vector. One adverse event that resulted in loss of vision in the treated eye was reported for the trial NTC04418427. Preexisting antibodies against both AAV2 and AAV8 have been detected in some individuals, and may confer immunological responses at high doses. In addition, direct intravitreal delivery of a more diffusible anti-VEGF drug has resulted in vascular complications in patients who experienced a reduction in vision.^19,20^ These outcomes have raised concerns that too much VEGF inhibition in the retina may result in undesired side effects. While the data suggest that long-term therapies for retinal vascular pathologies are feasible,^18^ choosing a suitable serotype that can elicit minimal immune responses and no side effects related to VEGF inhibition at therapeutic doses is an important factor to consider when designing an rAAV-based therapy for ocular vascular pathologies.

Several studies have described a wide range of transduced retinal cell types after intravitreal or subretinal delivery of either naturally occurring or engineered rAAV capsid.^21–24^ For example, AAV2.7m8, which is an engineered variant of the AAV2 serotype, has a much higher propensity to infect inner and outer retinal cells after intravitreal delivery than rAAV2.^21–24^ rAAV8, which was isolated from rhesus monkeys, is more efficient at transducing photoreceptors than rAAV2 following subretinal injections.^25,26^ A vector based on the AAV3b serotype was found to evade the human immune response seen against AAV2.^27^ However, its transduction of retinal cells is sporadic when delivered subretinally.^24^

Conbercept (KH902) is a recombinant protein that consists of multiple Ig domains of the vascular endothelial growth factor receptor 1 (VEGFR1) and VEGFR2, resulting in a high binding affinity protein to all VEGFA isoforms and other VEGF members.^28^ For clarity, we henceforth refer to the Good Manufacturing Practice manufactured drug as conbercept, while the recombinant transgene and its related rAAV-mediated protein products are referred to as KH902. Previous studies have shown that intravitreal delivery of conbercept inhibits angiogenesis in an oxygen-induced retinopathy (OIR) of prematurity model and promotes recovery of edemas in a laser damage model of CNV.^28–30^ A recent 1-year retrospective clinical study compared conbercept with another anti-VEGF drug in AMD patients with CNV. Conbercept was found to be significantly superior with respect to the number of treatments needed to reduce edemas.^31^ Furthermore, conbercept has been successfully applied to multiple retinal vascular diseases, such as diabetic retinopathy, as well as major and macular branch retinal vein occlusion.^32^

Here, we tested the efficacy and safety of an rAAV-mediated gene transfer strategy of KH902 to retinal cells in preventing and/or mitigating retinal vascular pathologies. We used four different AAV serotypes (AAV2, AAV2.7m8, AAV3b, and AAV8) to deliver by intravitreal injection a transgene cassette that expresses KH902 from a ubiquitous promoter. Therapeutic efficacy was tested in the OIR and the laser damage mouse models, while safety was tested in normal adult mice. We found that all four vector serotypes were able to reduce the number of retinal aneurysms, which are enlargements of blood vessels, in the OIR model. The magnitude in aneurysm reduction correlated with the transduction efficiency of the vector serotype, with AAV2.7m8 and AAV2 being the two most efficacious vectors. Similar results were obtained in adult mice with the laser damage model. There, AAV2.7m8 was not only effective at preventing the formation of choroidal neovascular lesions after laser damage, but was also able to reduce the number of active leakage sites and the overall surface area of neovascular lesions. As for safety, we found that high titers of rAAV2.7m8 and rAAV2 expressing KH902 resulted in a vascular sheathing pathology that is associated with immune cell infiltration. A similar pathology has also been reported in patients receiving anti-VEGF treatment who experienced a worsening in vision^3,19,20^ (NCT04418427). We show that this phenotype is associated with increased expression of intercellular adhesion molecule 1 (ICAM1) and vascular cell adhesion molecule 1 (VCAM1), two proteins that are essential for extravasation of immune cells by increasing vessel wall interaction with immune cells. This process, which has been well characterized in tumor angiogenesis,^33–35^ has been shown to be inhibited by VEGF. While the vascular sheathing pathology is prevented in immune-deficient Rag-1 mice that lack B and T cells, increased expression of ICAM1 in particular, and to a certain extent of VCAM1, is still seen, indicating that these changes are dependent on the inhibition of VEGF function and are not the result of an immune response. Finally, we show that this vascular sheathing pathology is dependent on the expression levels of KH902 as a relatively lower viral dose of rAAV2.7m8-*KH902* does not induce any vascular pathology, while it still efficiently reduces the number of active leakage sites. Together, the data suggest that long-term anti-VEGF therapy using rAAV-directed transgene expression of KH902 in the retina is a safe and viable option for the treatment of retinal vascular diseases through a single administration of the therapeutic vector.

## Materials and Methods

### Animals

The C57BL6 and Rag-1 (Stock no. 2216) mice were purchased from the Jackson Laboratory. *^rod^Tsc2^–/–^* mice were generated as described previously.^36^ All animals were maintained at a 12 h-light/12 h-dark cycle with unrestricted diets. All procedures involving animals were in compliance with the Association for Research in Vision and Ophthalmology (ARVO) Statement for the Use of Animals in Ophthalmic and Vision Research and were approved by the Institutional Animal Care and Use Committees (IACUC) of the University of Massachusetts Medical School.

### Vector design and production

The *KH902* 1,659 nt ORF was synthesized and subcloned into a pUC57 plasmid by GenScript. EcoRI+Kozak sequence and MluI sites were engineered at the 5′ and 3′ flanks of the ORF, respectively, using the following primers: F: GAA TTC GCC ACC ATG GTC AGC TAC TGG GAC ACC G; R: ACG CGT TCA TTT ACC CGG AGA CAG GGA GAG GC. Polymerase chain reaction (PCR) amplicons generated using KOD Hot Start Master Mix (EMD MilliporeSigma; Cat no. 71842-3) were subcloned into the TOPO vector using the Zero Blunt TOPO PCR Cloning Kit (Thermo Fisher; Cat no. 45-003-1). The fragment was cut from the TOPO vector using EcoRI and MluI restriction enzymes (NEB; Cat no. R0101S and R0198S, respectively) and cloned into compatible restriction sites within the pAAV2-chicken beta actin (CBA) plasmid to yield the pAAV-*KH902 cis* plasmid. The pAAV2.7m8 *trans* plasmid was obtained from Addgene (no. 64839). Plasmids were verified by Sanger sequencing and diagnostic digests. All vectors described were produced by triple transfection in HEK293 cells and purified by cesium chloride density gradient ultracentrifugation.^37^

### Transduction of retinal-pigmented epithelial cells *in vitro*

The retinal-pigmented epithelial (RPE) cells (ARPE-19, adult retinal-pigmented epithelial cell line-19; and hTERT RPE-1, human telomerase reverse transcriptase immortalized retinal-pigmented epithelial cell) were grown in Dulbecco's modified Eagle's medium/F12 medium containing 10% fetal bovine serum (FBS), at 37°C, in a 5% CO2 atmosphere. At 100% confluence, cells were transduced with rAAV2.7m8-*KH902*, rAAV2-*KH902*, or rAAV2.7m8-*eGFP*, 10^5^ GC/cell, in the presence or absence of Ad at an MOI of 100:1. Cells were then switched to either 5% FBS or kept in 10% FBS. After 72 h, media were collected and subjected to standard Western blot analysis using the human VEGFR1/Flt-1 biotinylated antibody (0.2 μg/mL, no.BAF321; R&D Systems), streptavidin-HRP (1:200 dilution; R&D Systems), and ECL WB substrate (Pierce).

### Human umbilical vein endothelial cell tube formation and cell proliferation assays

Human umbilical vein endothelial cells (HUVECs) (2 × 10^5^ cells/mL) in EBM-2 Endothelial Cell Growth Medium (CC-3162; Lonza) were treated with *conbercept* drug or 10-fold diluted condition media as detailed in the main text and 2 × 10^4^ cells were plated onto Matrigel (A1413202; Thermo Fisher)-coated 96-well plates and incubated for 30 min at 37°C. Afterward, 25 ng/mL of rhVEGF was added and cells were incubated for 12–24 h. Images were then acquired on a Leica microscope (DMI6000 B; Leica Microsystems) at 50 × magnification. The number of formed tubes per field was counted using ImageJ.

HUVECs (2 × 10^3^ cells) in EGM-2 were plated into 96-well plates and grown for 20–28 h. *Conbercept* drug or conditioned media as described were diluted 1:10 with EGM-2 medium, mixed with 25 ng/mL rhVEGF, and incubated at 37°C for 2–3 h. HUVECs were then incubated with the complex for 90–96 h. Ten microliters of cell counting kit-8 (CCK-8) solution (ApexBio; Cat no. K1018) was added to each well of the plate and incubated at 37°C for 2–4 h. Wells were mixed gently and absorbance was measured at 450 nm using a Synergy HT Microplate Reader (BioTek).

### Intravitreal delivery of rAAV vectors and KH902 protein

Intravitreally injections were performed as previously described^38^ in P0-P1 or adult mice as indicated in the text for OIR and laser damage models, respectively. Injections were performed with glass needles (Clunbury Scientific LLC; Cat no. B100-58-50) using the FemtoJet from Eppendorf with a constant pressure and injection time of 300 psi and 1.5 s, respectively, to deliver ∼1 μL of fluid into the vitreous. All viral concentrations were adjusted to 3 × 10^12^ vg/mL unless a specific dilution is noted in the text. The rAAV-*KH902* vectors were spiked with a 1:5 dilution of their corresponding rAAV-*eGFP* controls to verify that injections were delivered and distributed appropriately. The dilution was such that the final concentration of the rAAV-*KH902:eGFP* mixture was 3 × 10^12^ vg/mL. For the OIR model, the same mouse was injected in the right eye with the therapeutic vector and in the left eye with the control vector. For the laser damage model, mice were injected either with the rAAV-*KH902* vector or the corresponding control vector. The *conbercept* drug was injected using the same tools and approach (same volume) as the vectors at a concentration of 10 mg/mL per injection.

### OIR and laser damage model

Mice were injected at P0-P1 and caged with the nursing mom, incubated in a 70% of oxygen chamber (purchased from CoyLab; Cat no. 8430015) between P7-P12, and thereafter returned to normoxic environment (21% oxygen) until P18. Laser damage was performed with the TX-System from IRIDEX (532 nm laser) using the following setting: 100 μm diameter, 100 mW power, and a duration of 10 ms. Each mouse eye received 4–8 laser burns distributed across the retina. Handling of mice as well as fundus fluorescein angiography (FFA) and optical coherence tomography (OCT) (MICRON IV from Phoenix Technology Group) was performed as described previously^39^ to track damage sites at the time of damage and every 5 days thereafter as indicated in figures.

### Electroretinography

Electroretinograms (ERGs) were performed with the Celeris System (Diagnosys LLC) and their preset programs for scotopic and photopic recordings. Data shown were recorded with the following parameters. Scotopic recordings were performed at 1 cd.s/m^2^. Photopic ERG recordings used a background intensity of 9 cd.s/m^2^ and a flash intensity of 10 cd.s/m^2^. Handling of the animals was performed as previously described.^39^ Each group was composed of 10 eye recordings.

### Histology

Both cryosection and flat mount histology were performed as previously described.^38,39^ In brief, eye cups were dissected in cold 1 × phosphate-buffered saline (PBS) and fixed in 4% paraformaldehyde overnight at 4°C. Cryosections were cut at 12 μm thickness. For retinal and RPE flat mounts, the two tissues were separated before fixation in 4% paraformaldehyde.^39^ The following primary antibodies and dilutions were used: rat antiplatelet and endothelial cell adhesion molecule 1 (PECAM1) (1:300; NovusBio; Cat no. NB100-1642), rat anti-ICAM1 (1:300; Abcam; Cat no. ab119871), rabbit anti-VCAM1 (1:400; Cell Signaling Technology; Cat no. 39036), goat anti-hVEGFR1 (1:300; R&D Systems; Cat no. AF321), rat anti-MHC Class II (1:1,000; BD Pharmingen; Cat no. 556999), rat anti-CD4 (1:300; BD Pharmingen; Cat no. 553043), rat anti-CD41 (1:1,000; BD Pharmingen; Cat no. 553847), rabbit anti-Iba1 (1:300; Wako; Cat no. 019-19741), and rabbit anti-ZO1 (1:100; Invitrogen; Cat no. 40-2200). The following reagents had a chromophore conjugated: fluorescein peanut agglutinin lectin (PNA) (1:1,000; Vector Laboratories; Cat no. FL1071) and fluorescein Griffonia Simplicifolia Lectin I (GSL I) isolectin B4 (1:300; Vector Laboratories; Cat no. FL-1201). All antibodies were diluted in PBS with 0.3% Triton X-100 and 5% bovine serum albumin (Jackson ImmunoResearch; Cat no. 001-000-173). Nuclei were counterstained with 4′,6-diamidino-2-phenylindole (Sigma-Aldrich; Cat no. 9542). All secondary antibodies (1:500, donkey) were purchased from Jackson ImmunoResearch and were purified F(ab)2 fragments that displayed minimal cross-reactivity with other species. All images were visualized with a Leica DM6 Thunder microscope with a 16-bit monochrome camera. Surface area of leakage site and number of aneurysms were analyzed by IMARIS software using the surface area function.

### mRNA expression level

Retinal samples were collected as previously described.^40^ In brief, both retinas from the same mouse were pooled as one sample and RNA extraction was performed with TRIzol. Reverse transcription was performed with 500 ng total RNA using Quantabio qScript cDNA SuperMix (Cat. no. 95048) following the manual instructions. Multiplexed droplet digital polymerase chain reaction (ddPCR) was performed using a QX200 ddPCR system (Bio-Rad Laboratories, Hercules, CA) with TaqMan reagents targeting Kh902 and the reference gene, glucuronidase beta (GUSB) gene (no. 4448489; Thermo Fisher). Primer and probe sets for KH902 were designed and synthesized by Integrated DNA Technologies (Coralville, IA) (For.: 5′-GGACATACACAACCAGAGAGAC-3′; Rev.: 5′-GTGAGTGAAAGAGACACAGGAA-3, probe: 5′-/56-FAM/CCCATTTCA/ZEN/AAGGAGAAGCAGAGCCA/3IABkfq/-3′). *KH902* expression copy numbers were normalized to the GUSB gene expression copy numbers.

### Statistical analysis

Multiple *t*-test was used for two-group comparisons and two-way analysis of variance (ANOVA) for comparisons of more than two groups. Both analysis types were two-tailed. Significance levels were as follows: *p < 0.05; **p < 0.01; ***p < 0.001; and ****p < 0.0001. All bar graphs indicate mean and error bars represent the standard error of the mean. Denominators for graphs showing percentage of sites with active fluorescein leakage are indicated in figure legends. These graphs show the percentage of remaining sites that show fluorescein leakage at the days indicated. Error bars in these graphs represent margin of errors calculated with a 90% confidence interval.

## Results

### Vectorized KH902 inhibits VEGF function in retinal-pigmented epithelial cells *in vitro*

The *KH902* transgene coding sequence is 1,659 bp in size and produces a ∼70 kDa monomer. The mature protein is a 143 kDa dimer.^41^ Since the protein is secreted, cell-type-specific expression is not required. To maximize expression to achieve therapeutic levels of KH902 expression following transduction by intravitreal administration *in vivo*, we cloned the KH902 open reading frame into an AAV vector plasmid construct consisting of the cytomegalovirus enhancer, CBA promoter cassette, and the rabbit globin poly A sequence (Supplementary Fig. S1A). In addition, the consensus Kozak sequence was cloned 5′ of the translation start to promote efficient transgene expression. Vectors were first produced with the AAV2 and AAV2.7m8 capsids, the latter being a photoreceptor-tropic capsid discovered by directed evolution,^21^ to test proper expression of the transgene *in vitro*.

To determine whether rAAV-KH902 is expressed properly following vector transduction, we transduced the rAAV2- and rAAV2.7m8-KH902 vectors into cultured RPE cells (RPE-19 and hTERT-RPE) at an MOI of ∼100,000 virions per cell. After 72 h, the cell media were collected and subjected to Western blot analysis to assess the expression of KH902 (Supplementary Fig. S1B). We found that the 143-kDa dimer is properly expressed in RPE cells *in vitro* and is properly secreted as a dimer into the media. We note that cells infected with rAAV2-*KH902* consistently secrete more KH902 into the media than those infected by rAAV2.7m8-*KH902*, regardless of the percentage of FBS used in the experiment (RPE-19 and hTERT-RPE cells undergo terminal differentiation upon serum withdrawal) or whether cotransduced with an Ad helper virus, which improves transduction efficiency.

To demonstrate that the secreted protein was functional in blocking VEGF activity, we tested the capacity for the conditioned media to inhibit VEGF-mediated angiogenesis. The two hallmarks of angiogenic response to VEGF stimulation in cultured HUVECs are the formation of “tubes” and the induction of proliferation.^42,43^ We show that a 1:10 dilution of media conditioned by RPE-19 and hTERT-RPE cells transduced by rAAV2- and rAAV2.7m8-*KH902* can functionally and significantly reduce the frequency of tube formation to comparable levels such as 0.1 ng/μL of the *conbercept* drug (Supplementary Fig. S1C, D). CCK-8 assays also show that VEGF-induced proliferation can be reduced by the rAAV-*KH902* vectors at similar rates such as 0.1 ng/μL of the *conbercept* drug (Supplementary Fig. S1E). In contrast, conditioned media from cells transduced by rAAV2.7m8-*eGFP* had no effect in blocking tube formation or proliferation of HUVECs. Our data suggest that both rAAV2-*KH902* and rAAV2.7m8-*KH902* vectors can transduce RPE cells *in vitro* to produce and secrete functional KH902 in the media at levels that can effectively block VEGF-induced angiogenesis and proliferation.

### KH902 reduces the number of aneurysms in the OIR mouse model

**Figure 1.**
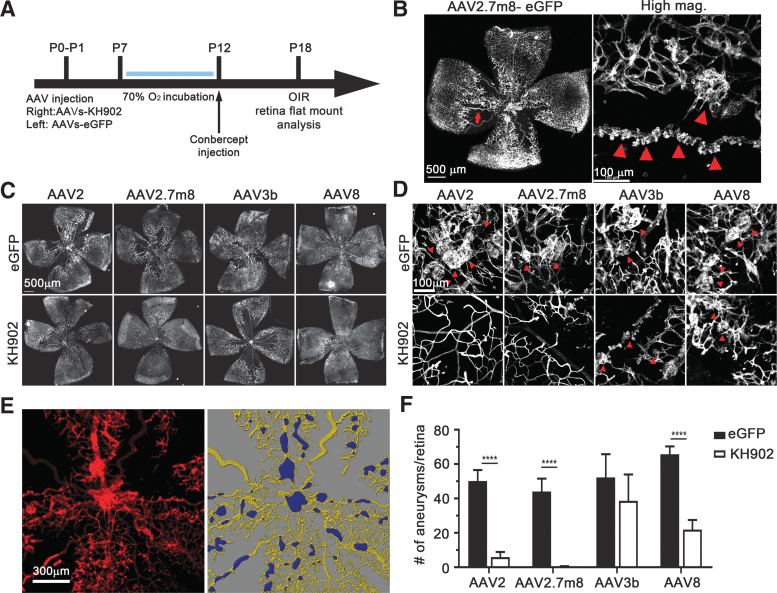
rAAV-*KH902* treatment reduces aneurysms in the OIR mouse model. **(A)** Time line of experimental design as described in the text for OIR. At P0-P1, the right eye was injected with the KH902 transgene and a 1:5 dilution of the *eGFP* transgene. The left eye served as control and was only injected with the vector carrying the *eGFP* transgene. All injections for any given litter used the same vector serotype. Mice receiving conbercept were injected at P12. **(B)** Representative retinal flat mount with aneurysms (*red arrow*). To the right: higher magnification of an area with several aneurysms (*red arrowheads*). Blood vessels and aneurysms were identified by PECAM1 staining (*white signal*). Scale bar = 500 μm (*left*) and 100 μm (*right*). **(C)** Representative retinal flat mounts stained with anti-PECAM1 antibody (*white signal*) from mice injected with the four different vector serotypes carrying either *eGFP* alone (*left eye: top row*) or the *KH902* transgene and the *eGFP* transgene (*right eye: bottom row*). Scale bar = 500 μm. **(D)** Higher magnification of abnormal vascularization seen in each treatment group shown in **(C)**. Aneurysms are indicated by red arrowheads. Scale bar = 50 μm. **(E)** Example of analysis performed with IMARIS software. Original PECAM1 staining (*red*) was masked (*yellow signal to the right*) and then in a central square of 1.2 × 1.2 mm, any surface area >20 μm^2^ was identified and counted as one aneurysm (*purple signal*) to determine the total number of aneurysms per retina. **(F)** Bar graph showing quantification of the average number of aneurysms per retina (*n* = 6–10 retinas) seen with the different serotypes. Shown is mean ± SEM (**p < 0.01, ****p < 0.0001). *eGFP*, enhanced green fluorescent protein; OIR, oxygen-induced retinopathy; PECAM1, platelet and endothelial cell adhesion molecule 1; rAAV, recombinant adeno-associated virus; SEM, standard error of the mean.

The OIR of the prematurity model is a well-established system for inducing abnormal vascular proliferation, which is characterized by a vascular catch-up growth phase that is induced by a preceding hyperoxic phase during the development of the retinal vasculature.^44^ Previous reports have shown that inhibition of VEGF during the catch-up phase by conbercept,^29^ or other anti-VEGF^45–47^ drugs, reduces neovascular areas in the OIR mouse model. We thus compared the efficacy of rAAV2- and rAAV2.7m8-*KH902* vectors in reducing neovascular pathologies in the OIR mouse model after intravitreal delivery. We also produced vectors using AAV3b and AAV8 serotypes (rAAV3b-*KH902* and rAAV8-*KH902*, respectively). Because expression of the transgene after viral infection takes several days, newborn pups were injected at postnatal days (P) P0-P1. An rAAV-*eGFP* vector harboring the same CBA promoter was used as control (left eye) and packaged with the same four capsid serotypes used to package *KH902* (right eye). At P7, the cage containing all pups of one litter with the mom was incubated in a 70% oxygen chamber for 5 days, and then returned to a normal oxygen environment (normoxia) until tissue was collected at P18 (Fig. 1A). Mice that were treated with the conbercept drug received an intravitreal delivery in their right eye at P12, immediately after removal from the 70% oxygen chamber (Fig. 1A). Because expression of *KH902* before P12 further dampens the development of the vasculature, and each vector serotype may exhibit different expression dynamics, we opted to quantify abnormal vascular growth by counting the number of aneurysms that form during the catch-up growth phase, rather than quantifying the area of normal vascularization, as has been done in other studies^45,46,48^ (Fig. 1B–D). Quantification of the number of aneurysms greater than 20 μm^2^ was performed in a 1.44 mm^2^ area centered on the optic nerve head, which is the most affected region (Fig. 1E). Staining the retina with an anti-PECAM1 antibody to highlight the retinal vasculature revealed aneurysms in all *eGFP* control-injected eyes (Fig. 1B–E). All four rAAV serotypes were able to reduce the number of aneurysms when compared with their respective *eGFP* controls (Fig. 1F). However, the reduction observed with rAAV3b-*KH902* was not statistically significant. The reduction in the number of aneurysms per retina seen with rAAV2-*KH902* and AAV2.7m8-*KH902* was greater than that seen by treatment with conbercept at P12 (Fig. 1F, Supplementary Fig. S2). In contrast, rAAV8-*KH902*, while showing a statistically significant reduction, still resulted in more than double the number of aneurysms per retina than what was achieved by conbercept treatment delivered at P12 (Fig. 1F, Supplementary Fig. S2).

To determine if the efficiency in reducing aneurysms correlated with the transduction efficiency of the vector serotype used, we examined the transduction of the four rAAV serotypes after intravitreal delivery in neonatal mice (Supplementary Fig. S3). Retinal flat mounts at P18 showed that rAAV2.7m8-*eGFP* resulted in the highest enhanced green fluorescent protein (EGFP) intensity followed by rAAV2-*eGFP* (Supplementary Fig. S3A). Retinas from mice injected with rAAV3b-*eGFP* had the lowest EGFP signal, while mice injected with rAAV8-*eGFP* had EGFP expression centered around the optic nerve head (Supplementary Fig. S3A). The results are consistent with the relatively higher numbers of aneurysms in the rAAV3b and rAAV8 groups. Section analyses showed that Müller glia were the predominant cell type transduced by all four vector serotypes (Supplementary Fig. S3B). In agreement with previous reports, rAAV2.7m8-*eGFP* also resulted in many EGFP-positive photoreceptors (Supplementary Fig. S3C). To better compare the transduction efficiency with the reduction in the number of aneurysms, we quantified the number of EGFP-positive cells per retinal flat mount (Supplementary Fig. S3D). While this method only provides a rough estimate of the number of transduced cells, as cells that are in different focal plains (*e.g.*, photoreceptors and Müller glial cells) cannot be properly resolved, the findings correlate with the data on aneurysm reduction (Fig. 1F, Supplementary Fig. S3D). rAAV2.7m8-*eGFP* resulted in the highest number of EGFP-positive cells (4.4 × 10^4^ cells) followed by rAAV2-*eGFP* (3.5 × 10^4^ cells). Both rAAV3b-*eGFP* and rAAV8-*eGFP* had fewer infected EGFP-positive cells. Interestingly, the number of EGFP-positive cells was similar between rAAV3b-*eGFP* and rAAV8-*eGFP* treatment groups, even though rAAV8-*KH902* conferred a better reduction in the number of aneurysms than was achieved by rAAV3b-*KH-902*. The propensity of rAAV8 to transduce cells mainly around the optic nerve head may explain this finding, as the quantification of the number of aneurysms was performed in the central region of the retina. Thus, localized secretion of KH902 protein in the central retina is likely higher in rAAV8-*KH902*-treated eyes than in rAAV3b-*KH902*-treated eyes. In all, the data show that vectorized KH902 can efficiently reduce the formation of aneurysms in the OIR mouse model. In addition, because the efficiency in aneurysm reduction is dependent on the transduction efficiency of the serotype used, our data suggest that the reduction in aneurysms is dependent on the dose of KH902 expression.

### KH902 reduces the occurrence of CNV in a prevention paradigm

To test the ability of the rAAV-*KH902* to prevent neovascular pathologies in the adult mouse, we used the laser damage model of CNV.^49^ This model uses a laser to damage the Bruch's membrane, which is the basal membrane onto which the RPE is attached. The other side of the membrane is abutted by the fenestrated choriocapillaris, which breaks through the damage site in the days following injury, mimicking CNV. To determine how much in advance the vector should be administered in a prevention paradigm model, we performed an expression analysis of KH902 transcript levels over time using ddPCR in mice injected with rAAV2.7m8-*KH902*. Over an 8-week time period, *KH902* transcript levels increased the most within the first week and then by a similar amount between weeks 1 and 4 (Supplementary Fig. S4A). Expression began to plateau after 4 weeks. Immunofluorescence analyses on retinal cross sections from eyes injected with rAAV2.7m8-*KH902* revealed that expression of KH902, as detected by anti-hVEGFR1 staining, was mainly seen in retinal ganglion cells (Supplementary Fig. S4B), even though EGFP expression was more prominent in Müller glial cells in rAAV2.7m8-*eGFP-*injected mice. A possible explanation for this finding is that Müller glial cells are more efficient at secreting the protein than retinal ganglion cells, thereby resulting in lower overall intracellular levels of KH902. We also quantified the number of EGFP-positive cells in adult mice after intravitreal delivery of the four different vector serotypes. As seen in neonates, rAAV2.7m8 injections resulted in the largest number of EGFP-positive cells, followed by rAAV2, rAAV3b, and rAAV8. Interestingly, rAAV8 exhibited a similar pattern of transduction as in neonatal retinas, where EGFP-positive cells were mostly centered around the optic nerve head. Similar to what was observed in eyes injected at neonatal stages, Müller glial cells were also the prominent cell type transduced by all four serotype vectors after intravitreal delivery in the adult eye (Supplementary Fig. S4C–E).

**Figure 2.**
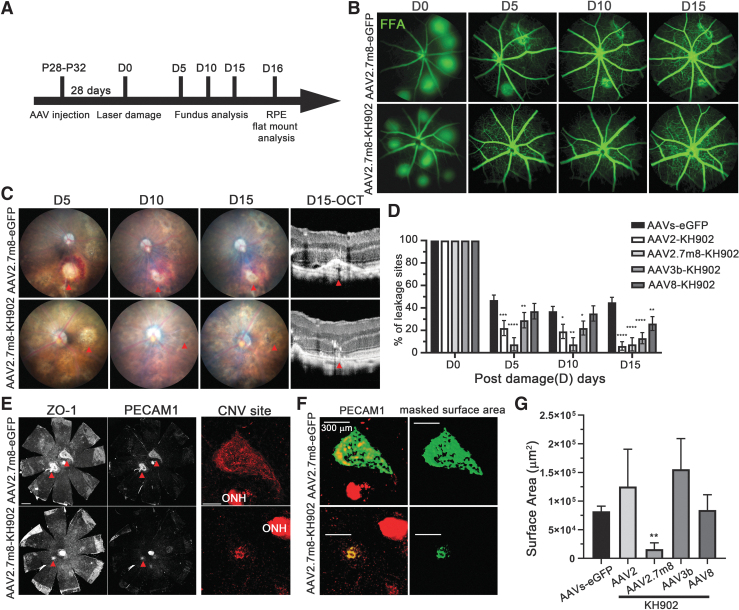
rAAV-*KH902* reduces laser damage-induced CNV in a prevention paradigm. **(A)** Time line of prevention paradigm as outlined in text. Laser damage **(D)** was performed on 4–8 different locations per retina (D0). Fundus and FFA were performed at D0 and over the next 15 days. **(B)** Representative FFA images of the same eye over time injected with vectors indicated. The number of leakage sites seen at D0 was used as denominator to calculate the percentage of remaining leakage sites over time. **(C)** Representative brightfield fundus images of the same eye over time injected with vectors indicated. Last column shows OCT analysis of CNV lesion at D15 (*red arrowheads* point to the same initial laser lesion). **(D)** Quantification of the percentage of the leakage sites remaining for each vector and time point indicated. The *eGFP* group shows combined data for all four *eGFP* vector serotypes. Number of initial leakage sites at D0: AAVs-*eGFP* (sum of all four *eGFP* serotypes) = 370, rAAV2-*KH902* = 102, rAAV2.7m8-*KH902* = 60, rAAV3b-*KH902* = 119, and rAAV8-*KH902* = 132. Bars show mean ± SEM (*p < 0.05, **p < 0.01, and ****p < 0.0001). **(E)** Representative RPE flat mounts stained for ZO-1 (ZO-1: RPE cell boundaries) and PECAM1 expression of eyes injected with indicated vectors, to identify neovascular lesions (*red arrowheads*). To the right, a higher magnification of PECAM1 staining (*red*) seen in the *middle column*, showing the remaining CNV sites. Scale bar = 500 μm (RPE flat mount) and 300 μm (CNV site) (ONH). **(F)** Example of output (*green signal*) generated by IMARIS software to quantify areas of CNV lesion. Scale bar = 300 μm. **(G)** Bar graph showing quantification of the average surface area of CNV lesion for serotypes indicated. AAVs-*eGFP* (average of all four *eGFP* serotypes). Values shown are mean ± SEM (**p < 0.01 and ****p < 0.0001). CNV, choroidal neovascularization; FFA, fundus fluorescein angiography; OCT, optical coherence tomography; ONH, optic nerve head; RPE, retinal-pigmented epithelial; ZO-1, zonula occludens 1.

To investigate how well rAAV can direct KH902 expression in the retina to prevent CNV, the rAAV-*KH902* or rAAV-*eGFP* vectors packaged with the four different serotypes were injected into 1-month-old mice (P28-P32). Because our temporal expression analysis (Supplementary Fig. S4A) showed that most of the increase in *KH902* transgene expression occurs in the first 4 weeks postinjection, laser damage was performed at 2 months of age. To determine how well CNV formation was prevented, we performed FFA immediately after laser damage and counted the total number of leakage sites that were induced. Since not all of the initial damage sites produce sufficient injury to the Bruch's membrane to produce a neovascular pathology, we reimaged the eyes 5 days postdamage to establish the total number of *bona fide* leakage sites that reflect neovascularization (Fig. 2A, B). Fundus and OCT imaging was used to confirm that the sites of active leakage at 5 days postdamage originated from the choroidal vasculature (Fig. 2C). Additional FFA imaging at 10 and 15 days postdamage was used to determine if the number of initial leakage sites counted at 5 days postdamage declined over time. The data show that with the EGFP control vectors, about 50% of all damage sites counted at day 0 were found to be vascularized by day 5 and remained so for the course of the experiment (Fig. 2D: rAAV-*eGFP* control vector showing the average of all four control serotypes). In contrast, eyes treated with vectors expressing KH902 generated with the AAV2, AAV2.7m8, and the AAV3b capsids had significantly <50% of leakage sites remaining at day 5, suggesting that they were able to prevent the initial formation of CNV. Only rAAV8-*KH902* was not able to prevent the formation of CNV pathology, although it was able to reduce leakage from choroidal neovascular sites over time (Fig. 2D). This reduction was not apparent with AAV2.7m8-*KH902*, likely because the initial prevention was so successful that the few sites that formed by postdamage day 5 must have suffered more severe damage and may thus be sites that take more time to heal. Interestingly, the number of active leakage sites at 15 days postdamage was similar between the rAAV2.7m8-*KH902* and rAAV2-*KH902* treatment groups; although at postdamage day 5, the difference seen between the two vectors was more than threefold. To further compare the data from the four vector serotypes, we quantified the choroidal neovascular surface area remaining at 15 days postlaser damage. We stained RPE flat mounts with a PECAM1 antibody to identify and quantify the size of the leakage sites by IMARIS software (Fig. 2E–G). We found that only treatment with rAAV2.7m8-*KH902* resulted in a clear reduction in the average size of the leakage sites when compared with its rAAV2.7m8-*eGFP* control. All other vectors did not show any reduction in the size of the CNV lesion when compared with their respective control groups or the combined average size of all *eGFP-*injected control mice (Fig. 4G; *eGFP* shows the average of all four serotypes). Together with the transduction efficiency data, these findings indicate that the vectorized form of KH902 can reduce the incidence of CNV and the leakage from neovascular pathologies in a prevention paradigm of the laser damage model. Furthermore, the data show that rAAV-*KH902* significantly reduces the growth of the neovascular lesions when using a serotype that exhibits high retinal transduction.

### Treatment-related vascular sheathing is mitigated by reduced *KH902* expression

**Figure 3.**
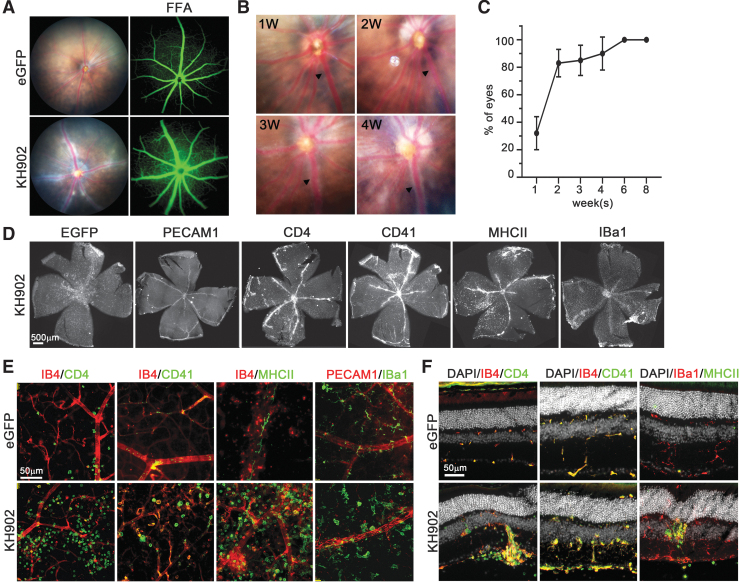
Vascular sheathing pathology seen in rAAV2.7m8-*KH902* transduced retinas. **(A)** Fundoscopy images showing white sheathing around major blood vessels in rAAV2.7m8-*KH902*-injected eyes. FFA does not show leakage of fluid from these vessels. **(B)** Representative images showing the development of the sheathing pathology over a 4-week time period. *Arrowhead* tracks the same vessel over time. **(C)** Quantification of the prevalence of the sheathing pathology over time (*n* = 18). Data are shown as percentage of eyes developing the pathology over time ± MOE. **(D)** Retinal flat mounts showing the distribution of different cell infiltrates in rAAV2.7m8-*KH902-*injected retinas. Cell markers are indicated above each panel. The EGFP panel shows the distribution of rAAV2.7m8-*eGFP* that was spiked into the rAAV2.7m8-*KH902* preparation at a ratio of 1:5. **(E)** Higher magnification of retinal flat mount images showing various cell infiltrates around the retinal vasculature in rAAV2.7m8-*KH902*-injected retinas (*bottom row*) when compared with control-injected retinas (*top row*). The different cell-type markers are indicated above each panel in the color depicted in the individual panels. Images are shown in *red/green* for better visualization even though they were acquired in Cy3 and Cy5. The green EGFP signal is not shown. **(F)** Same data as in **(E)** shown on retinal cross sections. Nuclear DAPI is shown in white. Scale bars in **(E)** and **(F)** = 50 μm. DAPI, 4′,6-diamidino-2-phenylindole; MOE, margin of errors.

Throughout the laser damage prevention paradigm, we observed a vascular sheathing pathology in mice injected with rAAV2.7m8-*KH902* and rAAV2-*KH902*, both of which resulted in high retinal transduction (Fig. 3A, Supplementary Fig. S5). The pathology was reminiscent of vasculitis, a vascular inflammation that is generally accompanied by immune cell infiltrates, although FFA imaging did not reveal any leakage of fluid from these inflamed blood vessels (Fig. 3A), a characteristic that is often seen in human retinas with vasculitis. To determine the frequency and dynamics of this pathology, we tracked mice injected with rAAV2.7m8-*KH902*, which displayed the highest transduction efficiency (Fig. Supplementary Fig. 4D), over an 8-week postinjection time period. At 2 weeks postinjection, over 80% of retinas had developed the sheathing pathology; and by 6 weeks, 100% of retinas displayed the pathology (Fig. 3B, C). To further investigate whether this sheathing pathology was caused by immune cell infiltrates, we used different cellular markers to determine the nature of the infiltrates (Fig. 3D–F). Some of the infiltrates were positive for PECAM1, which besides marking endothelial cells also marks platelets, macrophages, and lymphocytes. The strongest signal on retinal flat mounts, located mostly around major blood vessels, was seen with antibodies directed against CD4, CD41, and MHCII. CD4 marks a subpopulation of T cells, monocytes, and macrophages, while CD41 is an integral membrane protein expressed on platelets. MHC Class II marks glycoproteins on antigen-presenting cells that regulate the immune response by binding antigen-receptors on T cells (CD4^+^ T cells). Finally, we also used an antibody against IBa1, which marks macrophages, and ramified and activated microglia. We found many IBa1+ cells migrating from the inner plexiform layer to the ganglion cell layer and localizing around the main retinal blood vessels. Resident microglia of the inner and outer plexiform layers were also positive for IBa1. However, they were mostly ramified microglia and seen mainly in control-injected rAAV2.7m8-*eGFP* retinas. Taken together, the data show that the vascular sheathing is due to different immune cell infiltrates that migrate from the blood into the retina proper.

**Figure 4.**
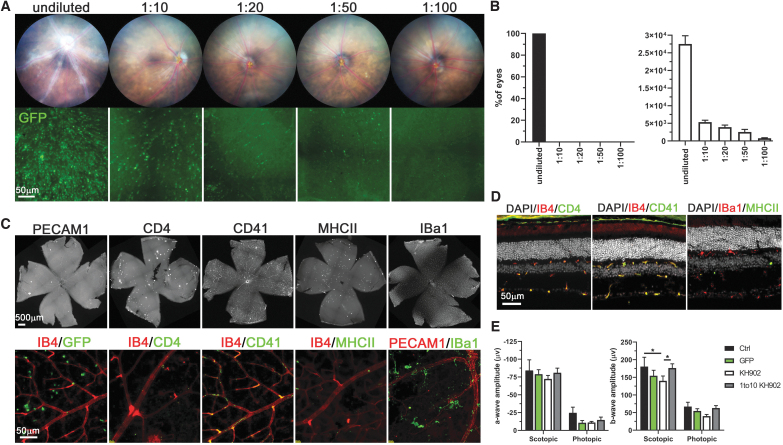
Dose-dependent effect of rAAV2.7m8-*KH902* on the vascular sheathing pathology. **(A)** Fundus images (*top row*) of eyes injected intravitreally with decreasing doses of rAAV2.7m8-*KH902*. *Bottom row* shows a higher magnification of EGFP signal seen on retinal flat mounts from eyes on *top row*. The rAAV2.7m8-*eGFP* vector was spiked at a ratio of 1:5 to the rAAV2.7m8-*KH902* vector to verify the dilution series. **(B)** Bar graphs showing the percentage of eyes that developed vascular pathology by 8 weeks postinjection (*left*) and the average number of EGFP+ cells per retina (*right*) at the different dilutions used. Results are shown as the percentage of eyes with sheathing pathology ± MOE (*left*), and the mean of the number of EGFP+ cells per retina ± SEM (*right*) (*n* = 8 eyes/dilution). **(C)** Retinal flat mounts of the 1:10 diluted rAAV2.7m8-*KH902*-injected eyes showing the distribution of the different cell markers indicated above each panel (*top row*). *Bottom row* shows a higher magnification of retinal flat mounts stained with the cell markers indicated above each panel in the color depicted in the individual panels. Images are shown in *red/green* for better visualization even though they were acquired in Cy3 and Cy5. The green EGFP signal is only shown in the first panel. Scale bar = 500 μm for flat mount and 50 μm for higher magnification. **(D)** Same data as in **(C)** shown on retinal cross sections. Nuclear DAPI is shown in *white*. Scale bar = 50 μm. **(E)** ERG recordings showing a-wave and b-wave amplitudes under scotopic and photopic conditions (*n* = 10 eyes/group). Mice were injected at P28 with vectors indicated, and ERG recordings were performed 8 weeks postinjections (controls are uninjected age-matched C57BL6 littermates at 12 weeks of age). Results are shown as mean ± SEM (*p < 0.05, **p < 0.01, and ****p < 0.0001). ERG, electroretinogram.

We next aimed to determine whether the vascular sheathing pathology was solely due to the high transduction efficiency of rAAV2.7m8-*KH902*, resulting in possibly excessive KH902 protein expression. A dilution series of the original titer was injected intravitreally and mice were analyzed at 8 weeks post-treatment, a time point when 100% of eyes injected with the undiluted rAAV2.7m8-*KH902* vector (3 × 10^9^ vg/eye) developed the sheathing pathology. To ensure proper injection and distribution of the vector, we spiked the rAAV2.7m8-*KH902* vector with the rAAV2.7m8-*eGFP* vector at a 1:5 ratio (*eGFP*:*KH902*) before performing the dilution series and the injections with the undiluted vector. Fundus analyses showed that a 1:10 dilution (3 × 10^8^ vg/eye) was sufficient to prevent any vascular sheathing from occurring (Fig. 4A, Supplementary Fig. S5). Transgene expression conferred by the dilution series was confirmed by quantifying the total EGFP-positive cell population on retinal flat mounts (Fig. 4A, B). To determine whether the absence of a visible fundus pathology also coincided with a reduction in immune cell infiltrates, we repeated the aforementioned immunostainings in the 1:10 dilution group. We did not detect any CD4^+^, CD41^+^, and MHCII+ cell infiltrates on retinal flat mounts and cross sections (Fig. 4C, D). The data suggest that immune cell infiltrates are partially driven by high KH902 expression and to a lesser extent by the capsid serotype since the undiluted rAAV2.7m8-*eGFP* alone elicits only a minimal immune response.

Loss of endogenous VEGF signaling in the eye has been associated with reduced visual function and retinal degeneration.^50,51^ To examine whether KH902 expression in the retina may affect photoreceptor functions, we performed ERG recordings to measure the a-wave and b-wave amplitudes of the cone and rod photopic and scotopic responses, respectively. Mice injected with 3 × 10^9^ vg/eye of rAAV2.7m8-*KH902* had a statistically significant reduction in the scotopic b-wave amplitude, when compared with age-matched uninjected mice or mice injected with a 1:10 dilution (3 × 10^8^ vg/eye) of the same vector (Fig. 4E). Interestingly, while mice injected with 3 × 10^9^ vg/eye of rAAV2.7m8-*eGFP* control vector had b-wave amplitudes that were better than 3 × 10^9^ vg/eye of rAAV2.7m8-*KH902* vector, they were not as good as the 1:10 dilution of the same vector. While these differences were not statistically significant, they do suggest that a high vector transduction may have some mild effect on retinal function. In agreement with this finding, we found some CD4^+^ cells in retinas injected with 3 × 10^9^ vg/eye of rAAV2.7m8-*eGFP* (Fig. 3E: first panel), while no CD4^+^ cells were seen in retinas treated with 3 × 10^8^ vg/eye of rAAV2.7m8-*KH902* or in uninjected age-matched mice (Fig. 4C, Supplementary Fig. S6). Photopic b-wave amplitudes, as well as photopic and scotopic a-wave amplitudes, showed a similar trend as scotopic b-wave amplitudes; however, the differences were not statistically significant (Fig. 4E). Together, the data suggest that a low vector load delivering the *KH902* transgene is potentially safer than a 10-fold higher vector load that delivers the *eGFP* transgene.

### Vascular sheathing pathology is associated with expression changes in cell adhesion molecules

**Figure 5.**
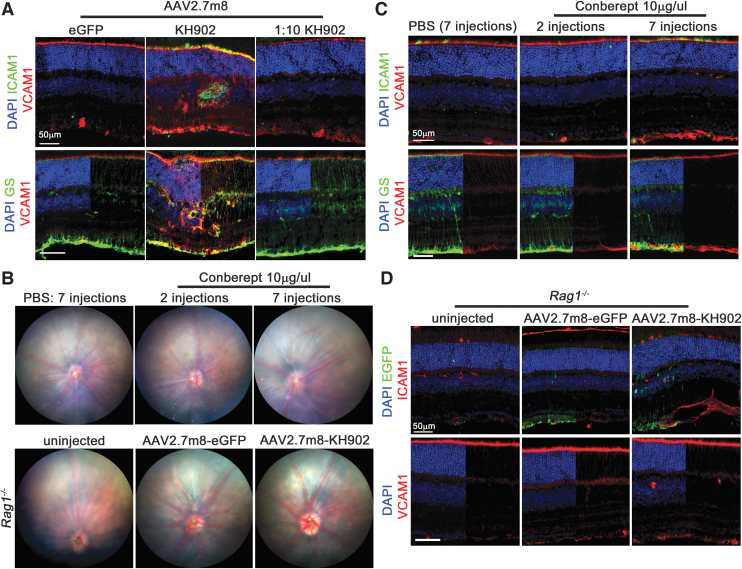
High dose of KH902 increases the expression of cell adhesion molecules. **(A)** Retinal cross sections of mice injected with vectors as indicated above each column. Increased expression of ICAM1 and VCAM1 is predominantly seen after injection of the undiluted rAAV2.7m8-*KH902* (*middle column*). **(B)** *Top row*: fundus images of mice injected with either PBS or two or seven injections of the conbercept drug (10 μg/injection). Images were taken at the end of the 2-week treatment. *Bottom row*: fundus images of Rag-1-deficient mice 1 month after injection of the vectors indicated. **(C)** Representative retinal cross sections as shown in **(A)** from mice shown in top row of panel **(B)**. Note, VCAM1 staining at the inner limiting membrane is more intense with seven injections of conbercept than what is seen with the 1:10 dilution of the rAAV2.7m8-*KH902* vector. However, ICAM expression is not increased in endothelial cells. A slight increase at the outer limiting membrane is seen close to the photoreceptor inner segments. **(D)** Representative retinal cross sections as shown in **(A)** from mice shown in bottom row of panel **(B)**. VCAM1 staining at the inner limiting membrane is slightly increased, while ICAM1 staining is significantly increased around endothelial cells as seen in wild-type mice. Panels **(A, C, D)** DAPI, *blue*; ICAM1, *green* in **(A, C)**; VCAM1, *red*; GS, *green* **(A)**; EGFP, *green* **(D)**. Scale bar = 50 μm. In some panels, the DAPI, GS, or EGFP signal has been removed from 50% of the panel to better visualize the remaining signal. GS, glutamine synthetase; ICAM1, intercellular adhesion molecule 1; PBS, phosphate-buffered saline; VCAM1, vascular cell adhesion molecule 1.

VEGF has been shown to inhibit the expression of ICAM1 and VCAM1 on endothelial cells.^33–35^ Both proteins play a pivotal role in the extravasation of immune cells from the blood circulation by increasing vessel wall interaction with immune cells.^33–35^ These interactions have been extensively studied in tumor angiogenesis.^33–35^ We thus investigated whether extravasation of immune cells was associated with increased expression of ICAM1 and VCAM1. In mice treated with 3 × 10^9^ vg/eye of rAAV2.7m8-*KH902*, we found increased expression of both ICAM1 and VCAM1. The increase in VCAM1 expression appeared to be mainly in Müller glial cells and vascular cells, with the most significant increase at the inner limiting membrane, where the first layer of the vascular plexus is located (Fig. 5A). The partial overlap of VCAM1 staining with glutamine synthetase, a Müller glial marker, at the inner limiting membrane suggests that additional cells such as astrocytes and vascular endothelial cells exhibit also increased VCAM1 expression. A similar expression pattern for VCAM1 has also been seen in noninfectious autoimmune uveitis.^52^ The increase in ICAM1 expression was seen mainly at the outer and inner limiting membrane, with a less pronounced staining at the inner limiting membrane than VCAM1 (Fig. 5A). In addition, there was a clear staining on endothelial cells in areas with immune cell infiltrates (Fig. 5A). These changes were not apparent in the rAAV2.7m8-*eGFP* control group or in mice that received 3 × 10^8^ of rAAV2.7m8-*KH902*, with the exception of a moderate increase of VACM1 at the inner limiting membrane region in mice injected with 3 × 10^8^ vg/eye of rAAV2.7m8-*KH902*.

To further determine whether the increased expression in endothelial cell adhesion molecules is directly associated with deleterious levels of KH902 protein, we performed intravitreal injections of the conbercept drug (10 μg/eye/injection) over a time period of 14 days. Two injection regimens were used: one regimen used two injections and the other, seven injections over the same time period. Fundus images at 14 days showed no vascular sheathing pathology in either of the two groups (Fig. 5B, top row); although by 14 days, more than 80% of eyes injected with 3 × 10^9^ vg/eye of rAAV2.7m8-*KH902* develop the sheathing pathology. However, we found increased expression of VCAM1 at the inner limiting membrane in mice receiving seven injections of conbercept. Importantly, this increase was more robust than the one seen in mice injected with 3 × 10^8^ vg/eye of rAAV2.7m8-*KH902*. Mice receiving two injections of conbercept or control mice receiving seven injections of PBS over the same time period did not show any increase in VCAM1 staining at the inner limiting membrane. Interestingly, ICAM1 expression was not altered with any of the injection regimens (Fig. 5C). Finally, to determine if KH902 causes gene expression changes in VCAM1 and ICAM1 that are driven by an immune response to the protein itself rather than by inhibition of VEGF function, we injected Rag-1-deficient mice^53^ with 3 × 10^9^ vg/eye of rAAV2.7m8-*KH902*. These mice lack B cells and T cells and are thus unable to mount any adaptive immune response. One month postinjection, we did not detect any vascular sheathing pathology by fundoscopy (Fig. 5B, bottom row). Flat mount analyses showed no CD4- or CD41-positive cells, and few MHCII-positive cells surrounding endothelial cells (Supplementary Fig. S7). With the exception of the MHCII-positive cells, the flat mounts resembled those injected with 3 × 10^8^ vg/eye of rAAV2.7m8-*KH902*. However, VCAM1 and ICAM1 expression was increased as seen with 3 × 10^9^ vg/eye of rAAV2.7m8-*KH902* in wild-type mice (Fig. 5D). In particular, ICAM1 expression was very pronounced in endothelial cells and at the outer limiting membrane, while VCAM1 showed more moderate increase at the inner limiting membrane (Fig. 5D). Taken together, data suggest that gene expression changes in VCAM1 and ICAM1 are independent of an immune response and likely driven in a dose-dependent manner by reduced VEGF function in the retina. Furthermore, the data suggest that the vascular sheathing pathology requires functional T and/or B cells to develop and that it is preceded by gene expression changes in VCAM1 and ICAM1 that promote extravasation of immune cells.

### rAAV-delivered KH902 reduces CNV after the onset of disease

**Figure 6.**
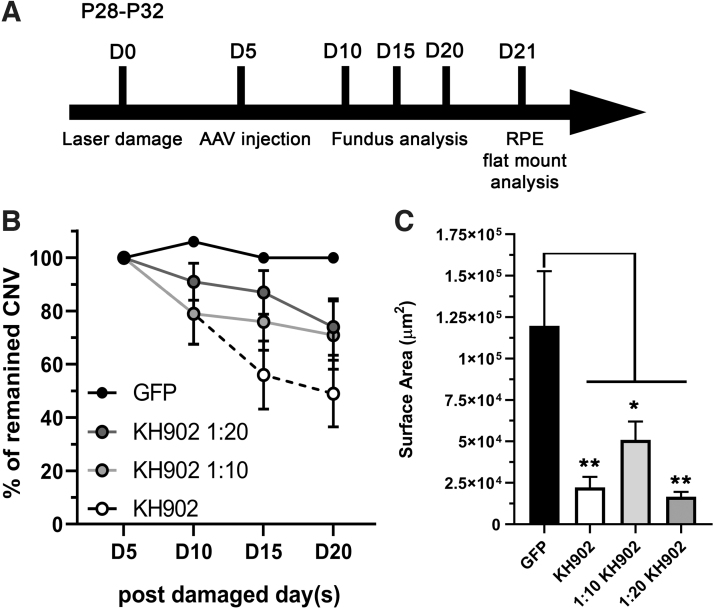
Treatment with rAAV2.7m8-*KH902* postlaser damage reduces CNV. **(A)** Experimental time line of laser damage model in a treatment paradigm. At the start of the laser damage (D0), mice were between P28 and P32. Five days later (D5), eyes were imaged by FFA to count the number of CNV lesions that developed in each treatment group (denominator). Subsequently, mice were injected intravitreally with the various vectors. Fundus, angiography, and OCT were performed over the next 15 days at D10, D15, and D20. RPE flat mounts were collected at D21 for histological analyses. **(B)** Graph showing percentage of remaining CNV leakage sites over time. Error bar = MOE (denominator: *n* = 30–45 leakage sites/vector at D5). **(C)** Surface area of CNV lesions, calculated as explained in Fig. 2, at D21 from RPE flat mounts stained with PECAM1 antibody. Results are shown as mean ± SEM (*n* = 30–45 lesion sites/vector).

Treatment of CNV in AMD patients occurs generally after the pathology has developed. We therefore examined in a treatment paradigm, whether vectored KH902 can also reduce choroidal blood leakage in eye with CNV. Because we found that lower doses of KH902 do not cause any vascular sheathing, and only a minimal increase in VCAM1 and ICAM1 expression, we also tested a 1:10 and 1:20 dilution of the original vector dose of 3 × 10^9^ vg/eye of rAAV2.7m8-*KH902*. Five days after laser damage, eyes were imaged by FFA to record the number of choroidal neovascular lesions that had developed. Immediately after imaging, mice were injected intravitreally with either 3 × 10^9^, 3 × 10^8^ (1:10) or 1.5 × 10^8^ (1:20) vg/eye of rAAV2.7m8-*KH902*, or 3 × 10^9^ vg/eye of the rAAV2.7m8-*eGFP* control vector (Fig. 6A). Over the next 15 days, mice were imaged by FFA in 5-day intervals to record the remaining percentage of active leakage sites (Fig. 6B). At the end of the experiment, we stained the RPE with PECAM1 to calculate the average size of the choroidal neovascular surface area that remained (Fig. 6C). We found a decrease in the percentage of active leakage sites over time with all three doses. Similarly, quantification of the choroidal neovascular surface area revealed also a significant reduction in the lesion size when compared with the rAAV2.7m8-*eGFP* control group. The data suggest that lower doses of vectored KH902 have still the potential to efficaciously treat CNV in humans.

## Discussion

rAAV vector-based anti-VEGF gene therapy has great potential for the treatment of retinovascular diseases. The sustained expression of the transgene product by retinal cells after a single administration overcomes the burden experienced by patients and the health care system from repeated injections of nongene therapy anti-VEGF drugs. However, possible inflammatory responses to the rAAV vector capsid, as well as undesired side effects from continuous VEGF inhibition, need to be carefully considered when developing such a strategy.

The goal of our study was to develop and test a novel anti-VEGF drug. We used four different rAAV vector serotypes to deliver the *KH902* transgene by intravitreal injection to retinal cells and examined its effect on retinovascular pathology using two different retinal vascular disease models, OIR and CNV, caused by laser damage. For the CNV model, we used a prevention and treatment paradigm. Our data show that vectored KH902 can be successfully used to treat retinovascular pathologies similar to conbercept injections.

We have previously shown that the photoreceptor transduction profile with subretinal rAAV injection is more dependent on the development of the photoreceptor outer segment than on the serotype used.^24^ Here we found that while the four rAAV vectors used (AAV2, AAV2.7m8, AAV3b, and AAV8) result in various transduction efficiencies with regard to cell number, cell type, and regional distribution, they all transduced Müller glial cells after intravitreal delivery in neonatal and adult mice. Given that Müller glial cells form the inner limiting membrane that abuts the vitreous, their efficient transduction by vitreal injection of rAAVs is not surprising. In the retina, VEGF is expressed in multiple cell types, including Müller glial and RPE cells.^54^ Müller glial cells also radially insert their processes between the retinal neurons and the retinal blood vessels forming a type of secondary retinal/blood barrier.^55–58^ They support the retinal structure and maintain retinal homeostasis.^55^ Thus, a high transduction of Müller glial cells expressing KH902 has the advantage to neutralize VEGF close to the sources of origins, and in proximity to retinal blood vessels. Interestingly, we found that KH902 accumulation was predominantly seen in retinal ganglion cells (Supplementary Fig. S2B) rather than in Müller glia. This may reflect the fact that Müller glia are naturally secretory cells, secreting various growth factors including VEGF.^59^ Thus, steady-state levels of KH902 in Müller glia might have been below the detectable threshold of the antibody, explaining why we were able to detect the protein in ganglion cells but not in Müller glial cells. While other rationales cannot be excluded to explain this finding at present, it is favored, as the transduction efficiencies of Müller glia by the four different vector serotypes appear to also correlate well with the treatment efficacies. In that regard, AAV8, which transduced Müller glia at high densities around the optic nerve head in the adult, performed less efficiently than AAV3b, as the total number of transduced Müller glia was low and too localized to the center of the eye. In contrast, the same central transduction profile of rAAV8 in the developing retina allowed rAAV8-*HK902* to perform better than AAV3b-*KH902* in the OIR model, since the quantification of aneurysms was performed in the center of the retina. A likely reason why AAV2.7m8 outperformed all other vectors is that even at a low dose, it was still able to efficiently and uniformly infect Müller glia across the retina after one intravitreal injection. The dose-dependent effect is also highlighted by the fact that in the prevention model of CNV, rAAV2.7m8-*KH902* was the only vector to prevent excess growth of neovascular lesions, as seen by the significantly smaller surface area of the lesions. Similarly, it also prevented excess growth of the neovascular lesion in the treatment paradigm. However, the experimental design in its current form cannot distinguish between whether the smaller lesion size is due to an actual reduction of the size or, rather, a result of growth prevention once KH902 expression starts. Additional data would be required to understand the kinetics of lesion growth during the onset of KH902 expression.

Inflammatory complications after intravitreal delivery of anti-VEGF drugs have been documented for all drugs currently in use.^3^ Recent clinical findings reported the occurrence of ocular vasculitis with associated decrease in vision in some wet AMD patients who received an intravitreal injection of a novel anti-VEGF antibody that is smaller and presumably may diffuse better to the back of the eye.^19,20^ A clinical trial (NCT04418427) that used rAAV2.7m8-*aflibercept* for the treatment of diabetic macular edemas reported a severe adverse inflammatory event in one patient that resulted in loss of vision in the treated eye. In our study, we observed a vasculitis-like phenotype in mice injected with rAAV2.7m8-*KH902* and rAAV2-*KH902*. We show that this phenotype correlates with the potency of rAAV-*KH902* transduction in adult mice. Furthermore, we show that the vascular sheathing pathology is associated with immune cell infiltration that is preceded by changes in VCAM1 and ICAM1 expression. Mice deficient in B and T cells do not develop a vascular sheathing pathology and thus lack associated immune infiltrates, yet they still upregulated VCAM1 and ICAM1 expression. This suggests that changes in gene expression that promotes extravasation of immune cells are a direct consequence of suppressed VEGF function, rather than an immune response to the KH902 protein. In tumor angiogenesis, VEGF has been found to reduce the expression of TNF-α-induced endothelial cell adhesion molecules, VCAM1 and ICAM1.^60^ Thus, too much reduction in native retinal VEGF may have deleterious consequences for the maintenance of retinal homeostasis. Blockage of VEGF signaling in the RPE has been shown to affect RPE, photoreceptor, and choriocapillaris health.^50,61^ Deletion of VEGFA in some retinal neurons during development has been shown to affect normal vascular development.^62^ A neuroprotective role for VEGF in photoreceptors, Müller glia, as well as ganglion cells has also been proposed,^51,63,64^ although overexpression of VEGFA binding proteins in photoreceptors appears not to affect retinal function^65^ nor does deletion of VEGF in Müller glial cells.^66^ While there is still controversy surrounding the risks associated with VEGF inhibition in patients,^67^ inflammatory complications in a small percentage of individuals are a well-documented side effect of the treatment.^3^ Even though most of these inflammations resolve spontaneously or can be managed with corticosteroids,^3^ meaning anti-VEGF drugs are safe for the management of ocular vascular pathologies, the fact that some type of inflammation is seen with all anti-VEGF drugs indicates that this effect is likely a consequence of VEGF inhibition.

We have shown that VCAM1 and ICAM1 expression increases upon injection of conbercept or rAAV-mediated gene transfer of KH902, even in immune-deficient Rag-1 mice. Both proteins help with the extravasation of immune cells from the vasculature. The different types of inflammations with different drugs and the contradictory findings from animal studies may thus be explained by the amount of anti-VEGF drug that actually remains functional in the retina for prolonged periods of time. For example, one intravitreal injection of the conbercept drug has been found to reduce serum VEGF levels by almost 90% 1 day postinjection.^68^ This means that most of the anti-VEGF drug must have diffused through the retinal vasculature into the main circulation. Thus, the amount that remains in the retina to inhibit retinal VEGF function might be rather small. Similar findings were reported for aflibercept, but not for ranibizumab.^69^ The difference between these drugs is that conbercept and aflibercept are fusion proteins of VEGFR1 and 2 that are fused to the constant Fc domain of human IgG1, while ranibizumab is a monoclonal antibody that likely has a lower binding affinity to VEGF than the native receptor domains. Beovu, which has been reported to cause vasculitis in one study,^3,19,20^ is a single chain antibody. Its smaller size may allow it to better diffuse to retinal cells, causing a stronger loss of retinal VEGF signaling. In the case of viral vectors, the cell type that produces the anti-VEGF drug is likely to be a determining factor. From a therapeutic perspective, Müller glia might be the best cells to target; however, overexpression of an anti-VEGF transgene in Müller glia may also cause more damage as Müller glia are naturally secretory cells. The observation that we were not able to detect KH902 in Müller glial cells, but were able to detect it in retinal ganglion cells, and the fact that overexpression of VEGFA binding proteins in photoreceptors appears not to affect retinal function,^65^ strengthen the idea that the cell type producing the anti-VEGF drug plays a pivotal role in the safety and efficacy of a rAAV anti-VEGF gene therapy. Thus, overexpressing an anti-VEGF drug in retinal cells other than Müller glia may be safer but less effective, requiring a higher viral titer. One exception with regard to safety may be retinal ganglion cells, as they appear to be already affected by the injectable drugs such as ranibizumab and aflibercept.^64^ Inhibiting VEGF signaling at one of the sources of production, and by a cell type that naturally secrets growth factors such as Müller glial cells, is likely to be more effective. Especially, since Müller glia-derived VEGF has been shown to be essential for diabetes-induced vascular leakage.^66^ Thus, the current controversy in the data may stem from the different approaches and drugs used. In agreement with this, we found that the conbercept drug did not induce vasculitis when delivered seven times within 14 days; yet vectored KH902 was able to cause vasculitis in more than 80% of injected eyes within 14 days. The absence of an immune response to the capsid with the conbercept drug, in addition to the local concentration of the anti-VEGF drug and cellular source of origin, may also explain the difference in outcomes between the drug and the transduction. In this regard, the conbercept drug was not sufficient to cause ICAM1 gene expression changes on endothelial cells, while the vectored KH902 did cause increased ICAM1 expression on endothelial cells of both wild-type mice and Rag-1-deficient mice. This suggests that it may take more time and a higher local concentration of the anti-VEGF drug for ICAM1 expression to increase. A future direct side-by-side comparison of our anti-VEGF drug with aflibercept, both packaged in AAV2.7m8 using the same promoter, would also be helpful in understating the different outcomes, as the two drugs are very similar in their design.

Another important factor to consider is the preexisting inflammation due to the disease condition. Most patients who suffer from AMD or diabetic retinopathy have already a certain degree of inflammation even without any edemas.^70,71^ Edemas further increase the inflammatory response as the immune system participates in removing excess fluid that has leaked into the neuronal tissue.^70,71^ Injecting anti-VEGF drugs in such patients is likely to further exacerbate the inflammatory response due to changes in VCAM1 and ICAM1 expression. To test this idea, we inject the lower safe dose of 3 × 10^8^ vg/eye of rAAV2.7m8-*KH902* intravitreally into a newly developed mouse model of AMD^39^ that shows more pronounced microglial activation and thus retinal inflammation.^36^ Six weeks postinjection, when the vascular sheathing pathology is detected in 100% C57Bl6 eyes at the higher dose of 3 × 10^9^ vg/eye, and in 0% of eyes at the lower dose of 3 × 10^8^ vg/eye, 53% of eyes had developed a uniform vascular sheathing pathology along most major blood vessels (Supplementary Fig. S8). None of the nondiseased control littermates, nor any of the *eGFP-*injected mice at a 10-fold higher dose, developed a uniform vascular sheathing pathology. The data indicate that preexisting inflammation can be exacerbated by inhibition of VEGF function. This may explain the occurrence of adverse events that lead to a reduction in vision in some rare cases with the injectable protein drugs. This phenomenon may become even more pronounced with viral injections, as most viral capsids elicit some immune response, and retinal transfection results in a higher local concentration of the anti-VEGF drug. Thus, a viral dose that appears safe in nondiseased animals may still cause severe adverse events either acutely or over time, as seen in our experiments, in rare instances where disease inflammation is rampant.

Here, we show that transduction of retinal cells with rAAV-*KH902* can efficiently prevent retinal vascular pathologies at low vector doses, while at high vector doses, rAAV-*KH902* can induce a vascular sheathing pathology. In addition, we show that a lower vector dose causes less of an immune response than a higher vector dose of the rAAV-*eGFP* control vector. The data demonstrate that prolonged expression of KH902 through rAAV-mediated gene transfer of a low viral dose, with a serotype that infects the retina uniformly, can be used to safely treat patients with neovascular pathologies in the future through one single intravitreal injection. What remains to be determined is the cell type(s) that can cause vasculitis when transduced. Restricting expression to a safe cell type and to anti-VEGF protein levels that are sufficient to prevent the neovascular pathology from occurring without causing long-term retinal damage will also improve the safety profile of rAAV-mediated anti-VEGF gene therapies.

## Supplementary Material

Supplemental data

Supplemental data

Supplemental data

Supplemental data

Supplemental data

Supplemental data

Supplemental data

Supplemental data
