# Supplementary material for: Low-Dose Recombinant Adeno-Associated Virus-Mediated Inhibition of Vascular Endothelial Growth Factor Can Treat Neovascular Pathologies Without Inducing Retinal Vasculitis
Source: Hum Gene Ther. 2021 Jul 19;32(13-14):649–66. doi: 10.1089/hum.2021.132 (PMC8312021; doi:10.1089/hum.2021.132)
Supplement: Supplemental data [file Supp_FigS1.pdf]

Supplementary Information

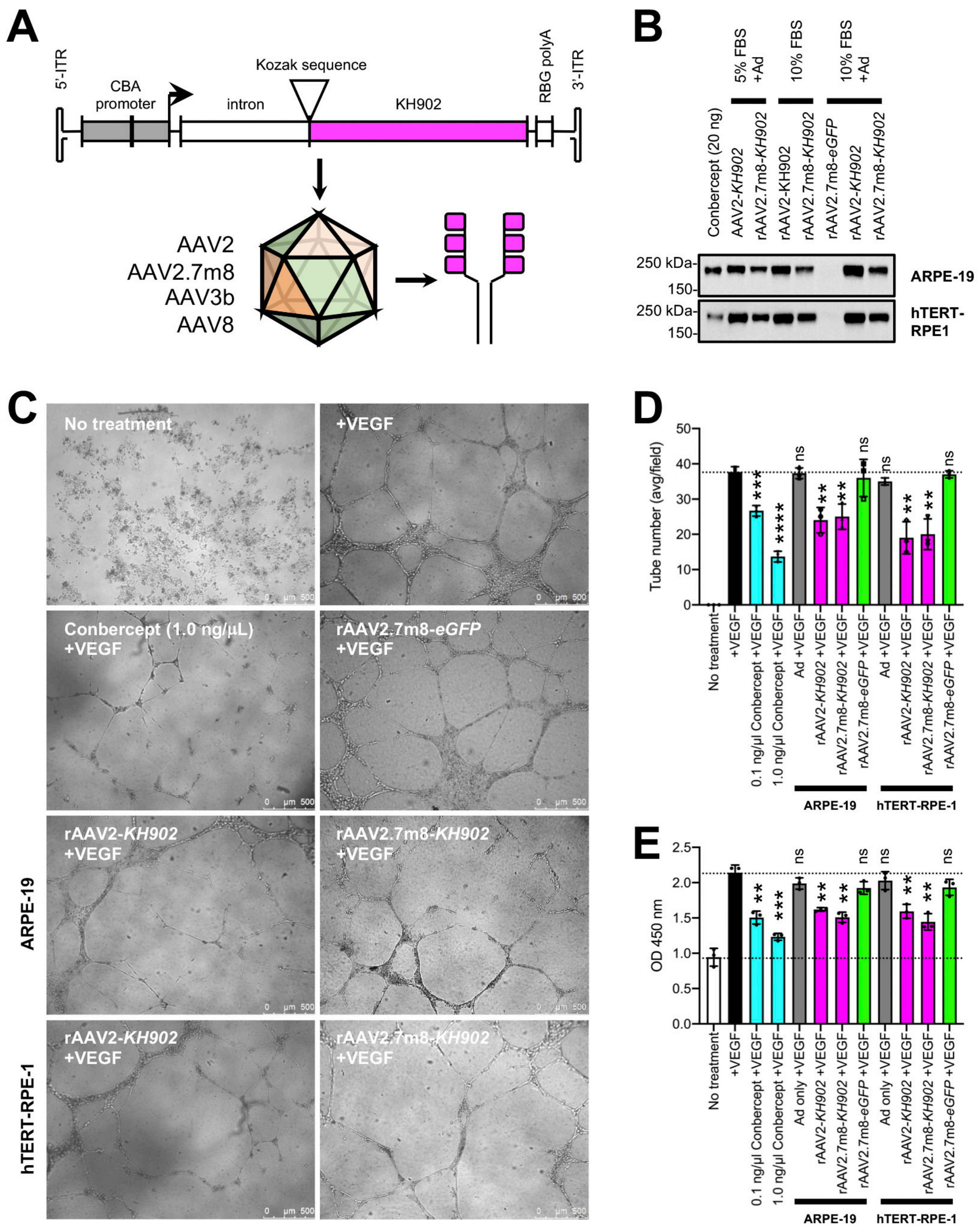

Fig. S1. Functional assessment of vectored KH902 by in vitro assays. **(A)** Diagram of the rAAV-CBA-KH902 construct. The rAAV vector expresses a secreted KH902 (conbercept) and is driven by the CMV enhancer and chicken β-actin promoter (CBA) cassette. A Kozak sequence was also designed 5' of the start codon to enhance translation initiation. Vectors produced for this study are packaged into AAV2, AAV2.7m8, AAV3b, and AAV8 capsids. The transgene product is a 143 kDa dimer. **(B)** Western blot analysis of conditioned media by cells (ARPE-19, top; and hTERT-RPE1, bottom) treated with the designated conditions for 72 hr. Membranes were subjected to blotting with anti-VEGFR1 antibody. 20 ng of the conbercept drug (first lanes) was included as reference for each blot. **(C, D)** In vitro functional validation of AAV-*KH902* vectors was assessed by quantifying the angiogenesis or the proliferative capacity of VEGF-stimulated (25 ng/mL) HUVECs while in the presence of conbercept drug; or conditioned media (diluted 1:10) of RPE cells infected with rAAV2-*KH902*, rAAV2.7m8-*KH902*, or rAAV2.7m8-*eGFP*. Anti-VEGF activity was quantified by tube formation assays (**C** and **D**). Panel (**C**) displays representative bright field images of HUVECs treated with cell-conditioned media and VEGF stimulation as described. Scale bars = 500 μm. Panel (**D**) represents quantification of average tubes formed per field. Anti-VEGF activity was also determined by CCK-8 activity (**E**), respectively. Values equal mean ±SD. ns, not significant; **, $p < 0.01$; ***, $p < 0.001$; ****, $p < 0.0001$.
