# Supplementary material for: Low-Dose Recombinant Adeno-Associated Virus-Mediated Inhibition of Vascular Endothelial Growth Factor Can Treat Neovascular Pathologies Without Inducing Retinal Vasculitis
Source: Hum Gene Ther. 2021 Jul 19;32(13-14):649–66. doi: 10.1089/hum.2021.132 (PMC8312021; doi:10.1089/hum.2021.132)
Supplement: Supplemental data [file Supp_FigS2.pdf]

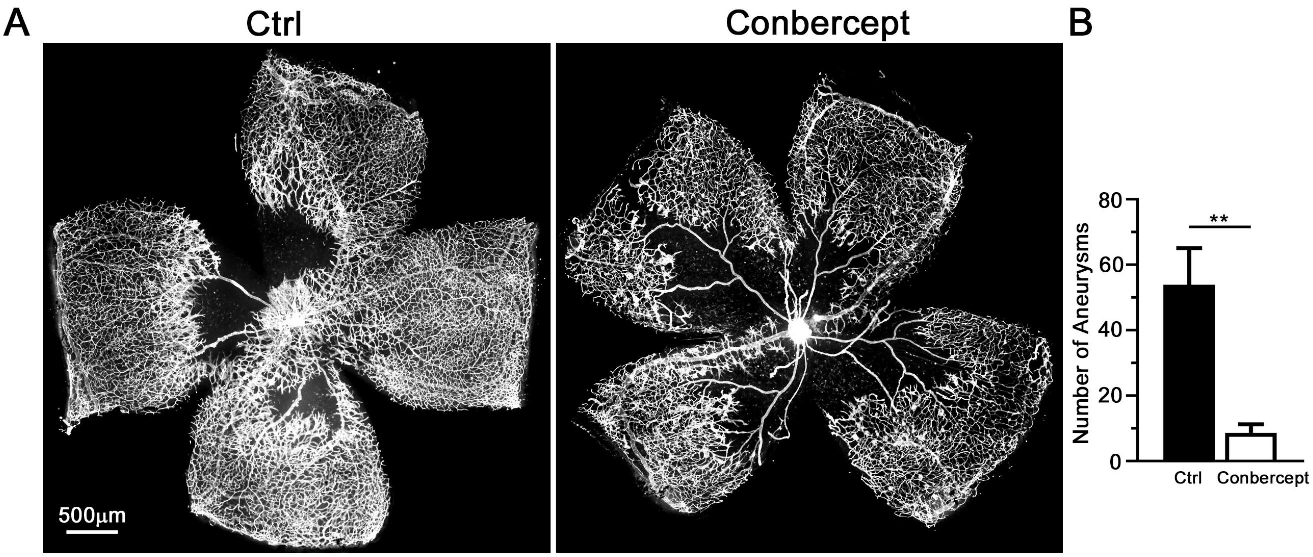

Fig. S2. Retinas treated with the Conbercept drug show a decrease in the number of aneurysms. **(A)** Representative images of OIR retinas that are either untreated (left) or treated with the conbercept drug (right) and stained with an anti-PECAM-1 antibody to examine vascular pathology. Treatment was performed at P12 by intravitreal injection of 1 μl of a 10μg/μl solution of the conbercept drug. Scale bar=500μm. **(B)** Quantification of number of aneurysms per retina. Bar graph shows mean ± S.E.M (n=6-10 retinas; ** $P < 0.01$, **** $P < 0.0001$).
