# Supplementary material for: Low-Dose Recombinant Adeno-Associated Virus-Mediated Inhibition of Vascular Endothelial Growth Factor Can Treat Neovascular Pathologies Without Inducing Retinal Vasculitis
Source: Hum Gene Ther. 2021 Jul 19;32(13-14):649–66. doi: 10.1089/hum.2021.132 (PMC8312021; doi:10.1089/hum.2021.132)
Supplement: Supplemental data [file Supp_FigS3.pdf]

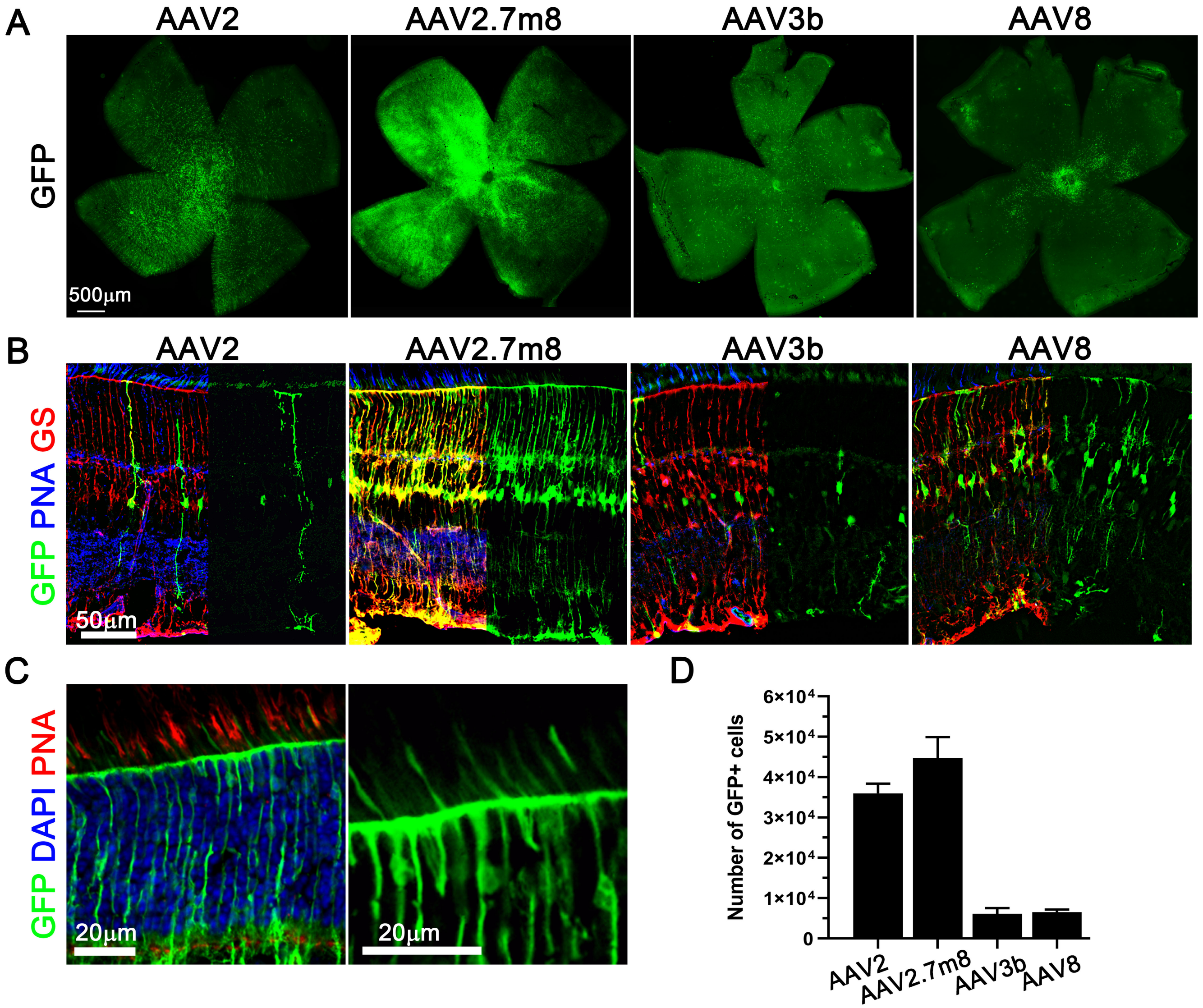

Fig. S3. Comparison of transduction efficiency of rAAV vectors after intravitreal injection in neonatal mice. **(A)** Retinal flat mounts showing EGFP expression (green) seen after intravitreal injection at P1 with the four different rAAV serotypes indicated above each panel. Scale bar = 500 µm. **(B)** Cryo-sections of retinas infected with vector serotypes shown in **(A)**. Green signal shows EGFP expression from viral infection. Sections were also stained with peanut agglutinin lectin (PNA: blue), which highlight cone segments, and an antibody against glutamine synthetase (GS: red), which marks Müller glial cells. Each image has the right half of the GS signal removed for better visualization of green signal. Scale bar = 50µm. **(C)** Higher magnification of retina infected with rAAV2.7m8-*eGFP* showing EGFP in the outer nuclear layer and photoreceptor inner segments (EGFP: green; PNA: red; nuclear DAPI: blue). **(D)** Quantification showing average number of EGFP+ cells per retina for each AAV vector serotype used. Results are shown as mean ±S.E.M. (n = 6-10 retinas/serotype).
