# Supplementary material for: Low-Dose Recombinant Adeno-Associated Virus-Mediated Inhibition of Vascular Endothelial Growth Factor Can Treat Neovascular Pathologies Without Inducing Retinal Vasculitis
Source: Hum Gene Ther. 2021 Jul 19;32(13-14):649–66. doi: 10.1089/hum.2021.132 (PMC8312021; doi:10.1089/hum.2021.132)
Supplement: Supplemental data [file Supp_FigS4.pdf]

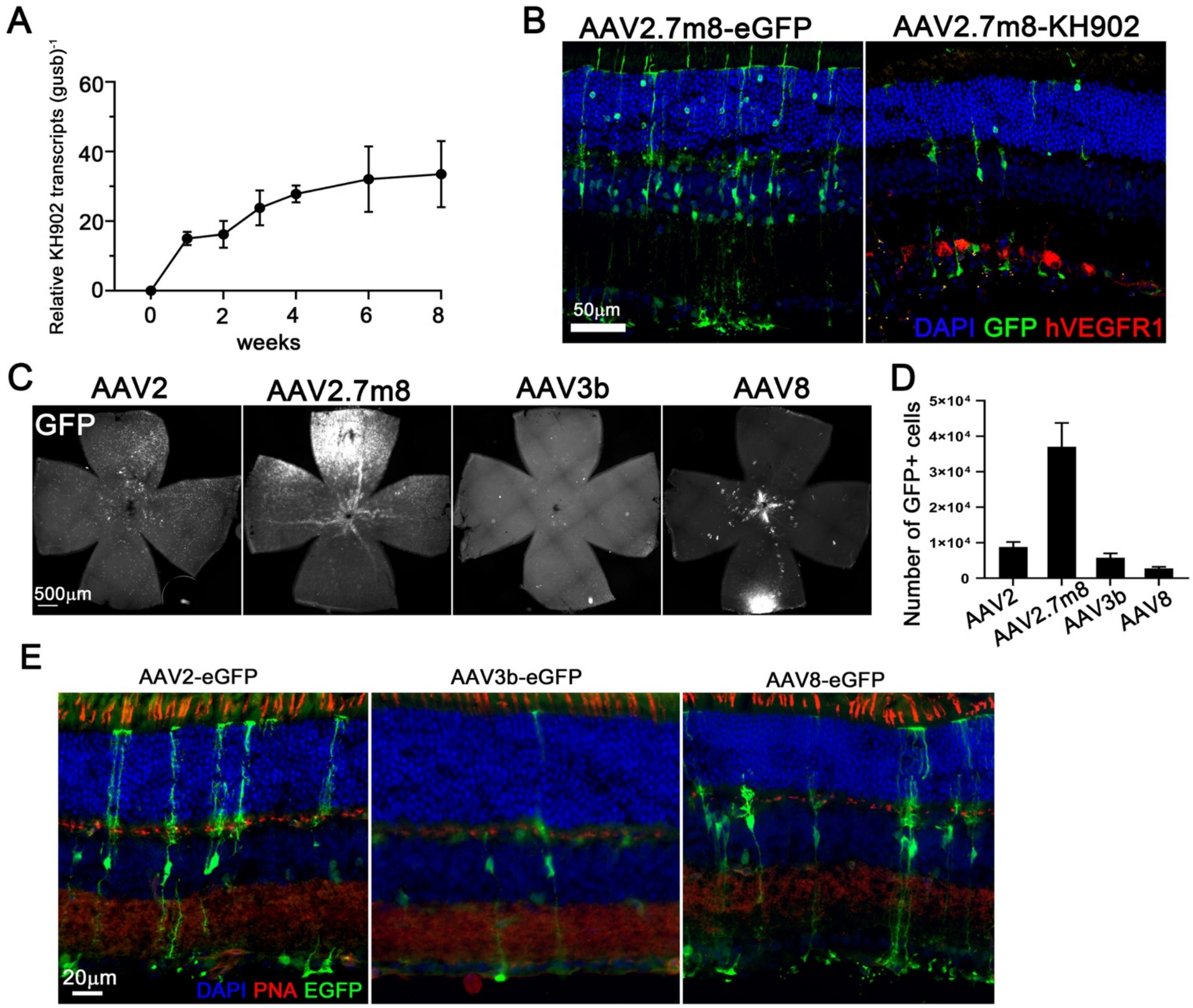

Fig. S4. Comparison of transduction efficiency of rAAV vectors after intravitreal injection in adult mice. **(A)** *KH902* mRNA expression levels over time in mice injected with AAV2.7m8-*KH902* (Error bar = S.E.M. n=3 retinas/time point). **(B)** Retinal cryo-section (right panel) of mice injected with AAV2.7m8-*KH902* and a 1 to 5 dilution of AAV2.7m8-*eGFP* (green signal) showing KH902 expression (anti-human VEGFR1 antibody, red signal) in ganglion cells. Left panel shows cryo-section of eye injected with AAV2.7m8-*eGFP* control virus alone. No hVEGFR1 signal is seen in those retinas (EGFP, green; DAPI, blue; hVEGFR1: red; Scale bar = 50μm). **(C)** Representative retinal flat mount images showing transduction patterns of each AAV vector serotype with the *eGFP* transgene (white signal) injected at P32. All 4 vectors show a broad distribution across the entire retina with the exception of AAV8-*eGFP*, which seems to infect mainly the region around the optic nerve head. The other 3 serotypes differ mainly in the density of EGFP+ cells as seen on flat mounts. Scale bar= 500μm. Note, bright signal at periphery seen with AAV8-*eGFP* stems for peripheral damage during injection. Because rAAV8 has a propensity to infect photoreceptors when injected sub-retinal, many photoreceptors are seen at the injection site as injections were performed through the choroid close to the iris. **(D)** Quantification of total number of EGFP+ cells per retina seen with each AAV vector serotype. Results are shown as mean ± S.E.M. (n=5 retinas). **(E)** Cryo-sections of eyes infected with the serotypes indicated. The predominant cell type transduced with each serotype are Müller glia cells.
