# Supplementary material for: Low-Dose Recombinant Adeno-Associated Virus-Mediated Inhibition of Vascular Endothelial Growth Factor Can Treat Neovascular Pathologies Without Inducing Retinal Vasculitis
Source: Hum Gene Ther. 2021 Jul 19;32(13-14):649–66. doi: 10.1089/hum.2021.132 (PMC8312021; doi:10.1089/hum.2021.132)
Supplement: Supplemental data [file Supp_FigS5.pdf]

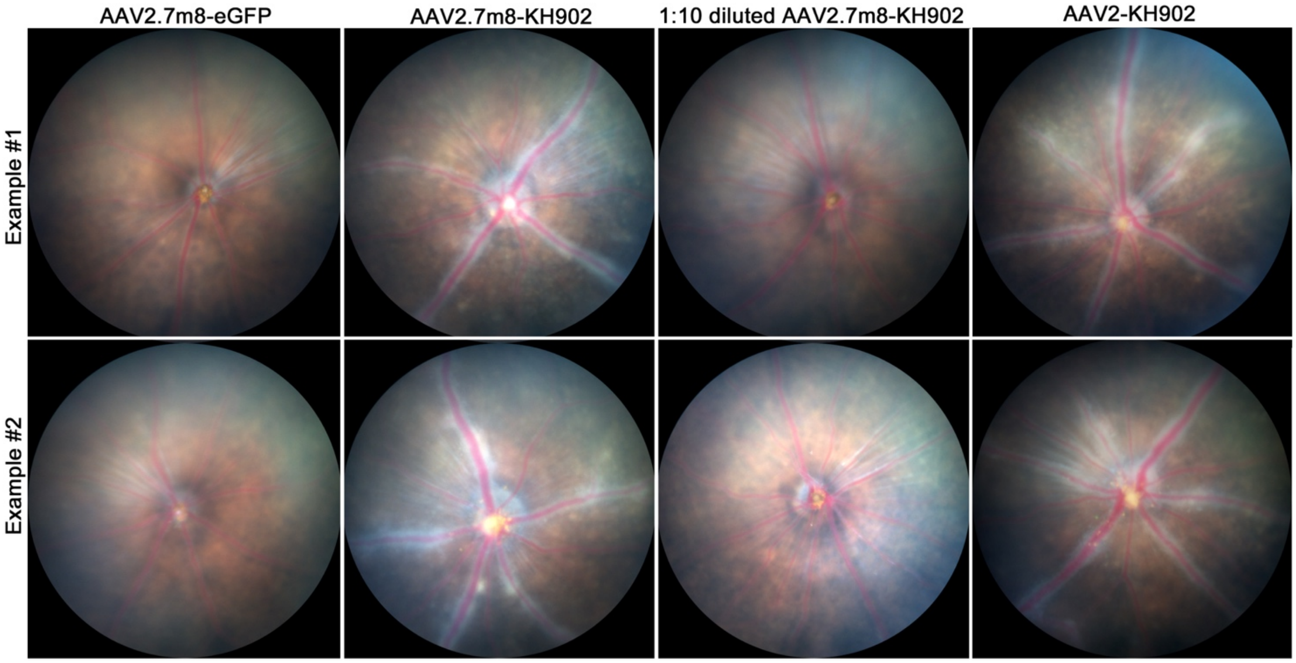


**Fig. S5.** Examples of vascular sheathing pathology. Fundus images of different eyes injected with AAV2.7m8-*eGFP*, AAV2.7m8-*KH902*, 1:10 dilution of AAV2.7m8-*KH902* and AAV2-*KH902*. Images were taken 8 weeks post injections.
