# Supplementary material for: Low-Dose Recombinant Adeno-Associated Virus-Mediated Inhibition of Vascular Endothelial Growth Factor Can Treat Neovascular Pathologies Without Inducing Retinal Vasculitis
Source: Hum Gene Ther. 2021 Jul 19;32(13-14):649–66. doi: 10.1089/hum.2021.132 (PMC8312021; doi:10.1089/hum.2021.132)
Supplement: Supplemental data [file Supp_FigS6.pdf]

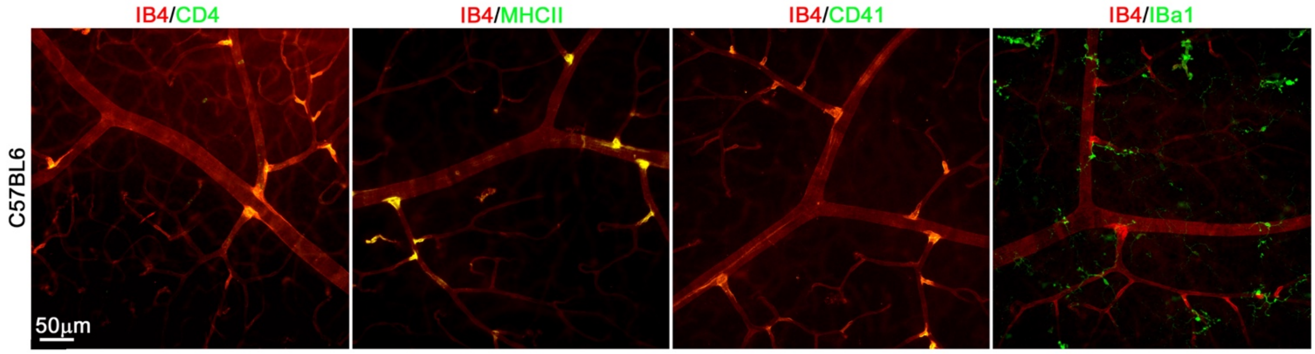

Fig. S6. Distribution of cell type markers in uninjected C57Bl6 control mice. Shown are higher magnification images of retinal flat mounts showing the distribution of the different cell infiltrates in uninjected C57Bl6 control mice. The different cell type markers used are indicated on top of each panel in the color depicted in the individual panels. Figure serves as control figure for Fig. 3E and Fig. 4C. Except for Iba1 positive cells in the correct location no cell infiltrates are seen in C57Bl6 mice.
