# Supplementary material for: Low-Dose Recombinant Adeno-Associated Virus-Mediated Inhibition of Vascular Endothelial Growth Factor Can Treat Neovascular Pathologies Without Inducing Retinal Vasculitis
Source: Hum Gene Ther. 2021 Jul 19;32(13-14):649–66. doi: 10.1089/hum.2021.132 (PMC8312021; doi:10.1089/hum.2021.132)
Supplement: Supplemental data [file Supp_FigS7.pdf]

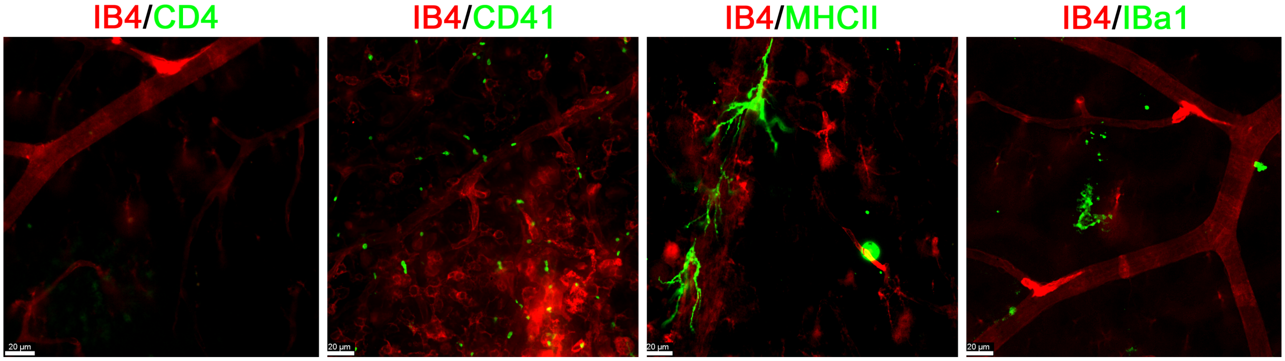

Fig. S7. Distribution of cell type markers in Rag mice injected with the undiluted ($3*10^9$ vg/eye) AAV2.7m8-*KH902*. Shown are higher magnification images of retinal flat mounts showing the distribution of the different cell infiltrates. The different cell type markers used are indicated on top of each panel in the color depicted in the individual panels. While CD4+ and CD41+ cells are not seen in Rag1 mice injected with AAV2.7m8-*KH902* there are MHCII positive cells that surround the retinal vasculature (IB4 signal in red). The figure is similar to Supplementary Fig. S6, and Fig. 3 and Fig. 4.
