# Supplementary material for: Low-Dose Recombinant Adeno-Associated Virus-Mediated Inhibition of Vascular Endothelial Growth Factor Can Treat Neovascular Pathologies Without Inducing Retinal Vasculitis
Source: Hum Gene Ther. 2021 Jul 19;32(13-14):649–66. doi: 10.1089/hum.2021.132 (PMC8312021; doi:10.1089/hum.2021.132)
Supplement: Supplemental data [file Supp_FigS8.pdf]

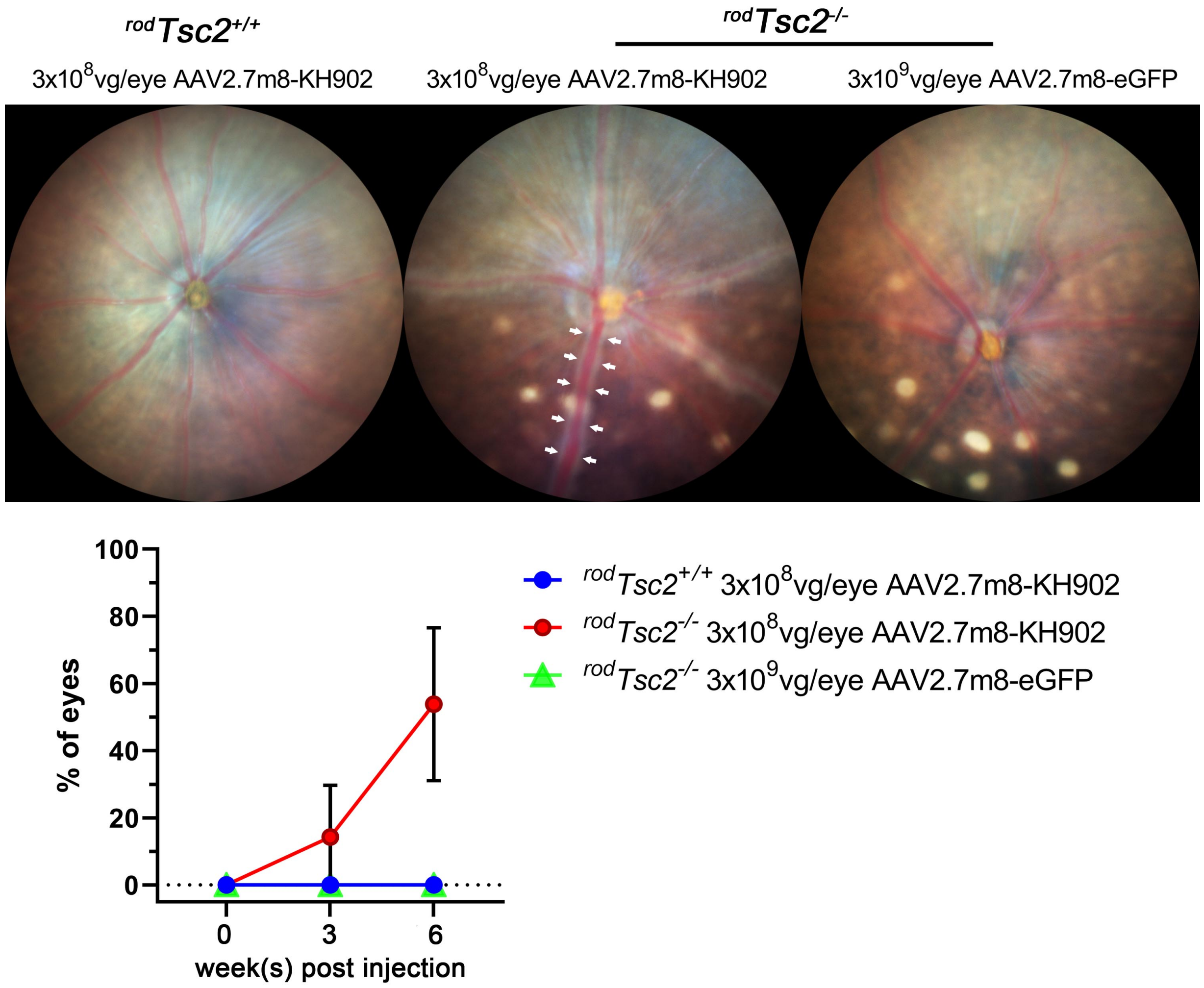

Fig. S8. Development of vascular sheathing pathology in a model of Age-related macular degeneration at 6 weeks post intravitreal injection. Top panels show examples fundus images in a mouse strain that develops AMD-like pathologies. Left panel: $^{rod}Tsc2^{+/+}$ mice injected with $3x10^8$ vg/eye of AAV2.7m8-*KH902*. No vascular sheathing pathology is seen in this mouse strain, similar to C57Bl6 injected with the same dose. Middle panel: $^{rod}Tsc2^{-/-}$ mice injected with $3x10^8$ vg/eye of AAV2.7m8-*KH902* develop a uniform vascular sheathing pathology (white arrows point to one of the blood vessel with vascular sheathing pathology) in approximately 50% of eyes. $^{rod}Tsc2^{-/-}$ mice and $^{rod}Tsc2^{+/+}$ mice were generated by crossing the *Tsc2*$^{c/c}$ mice with *Tsc2*$^{c/c}$ mice that also carry the *i75Cre* transgene, which drives CRE protein expression in rod photoreceptors (https://www.mdpi.com/2218-273X/11/6/871). The resulting CRE-negative mice are $^{rod}Tsc2^{+/+}$ mice, while the CRE-positive mice are $^{rod}Tsc2^{-/-}$ mice that develop AMD-like pathologies with inflammation. Right panel shows $^{rod}Tsc2^{-/-}$ mice injected with $3x10^9$ vg/eye of AAV2.7m8-*eGFP* control virus. Even at a 10-fold higher dose there is no vascular sheathing pathology with the control virus. Graph below shows the percentage of eyes that develop a vascular sheathing pathology over time. Eyes were imaged at 3 weeks and 6 weeks post-injection of the virus since by 6 weeks post-injection 100% of C57Bl6 mice develop a vascular sheathing pathology at the higher dose. $^{rod}Tsc2^{-/-}$ mice and corresponding control littermates ($^{rod}Tsc2^{+/+}$) were 3 months old at the time of injection.
